# Comorbidity patterns and mortality in HFpEF: A retrospective longitudinal cohort study

**DOI:** 10.1016/j.ijcrp.2025.200526

**Published:** 2025-10-10

**Authors:** Mohammed Yousufuddin, Zeliang Ma, Ebrahim Barkoudah, Muhammad Waqas Tahir, Ali Yazdanyar, Rani Chikkanna, Khalid Benkhadra, Sumit Bhagra, Gregg C. Fonarow, Mohamad H. Yamani

**Affiliations:** a Department of Hospital Internal Medicine, Mayo Clinic Health System, Austin, MN, USA; b Department of Hospital Medicine, Brigham and Women's Hospital, Harvard Medical School, Boston, and Baystate Health, Springfield, MA, USA; c Department of Hospital Internal Medicine, Mayo Clinic, Jacksonville, FL, USA; d Division of Hospital Medicine, Department of Medicine, Perelman School of Medicine, University of Pennsylvania, Philadelphia, PA, USA; e Hospital Medicine, Albert B. Chandler Medical Center, University of Kentucky, Lexington, KY, USA; f Department of Hospital Internal Medicine, Mayo Clinic, Phoenix, AZ, USA; g Department of Endocrine and Metabolism, Mayo Clinic Health System, Austin, MN, USA; h Division of Cardiology, University of California, Los Angeles, Los Angeles, CA, USA; i Department of Cardiovascular Medicine, Circulatory Failure, Mayo Clinic, Jacksonville, FL, USA

**Keywords:** Heart failure, Comorbidities, Mortality, Rural-urban, Sex, Age

## Abstract

**Background:**

Comorbid conditions (CCs) in heart failure with preserved ejection fraction (HFpEF) are associated with poor prognosis, but the influence of their duration on mortality remains unclear. We examined how pre-admission comorbidity duration affects long-term mortality after HFpEF hospitalization.

**Methods:**

This retrospective study included adults hospitalized for HFpEF at 17 hospitals (2010–2022) with follow-up through July 2024. Twelve individual and four system-based comorbidities present at admission were classified by duration: new (<0.5 years), early (0.5–<3 years), intermediate (3–<6 years), and long-term (>6 years). Mortality was further stratified by Get With The Guidelines–Heart Failure (GWTG) score (1, 2, ≥3) and discharge SGLT2i prescription. Cox regression and restricted cubic splines estimated risk.

**Results:**

Among 9256 patients (mean age 77.8 years; 54.1 % female; 40.2 % rural), 64.1 % died over a median follow-up of 2.7 years. Comorbidity prevalence ranged from 6.0 % (neurological) to 73.4 % (hypertension), with durations from 0.5 years (obesity) to 5 years (diabetes). Most comorbidities increased mortality risk (HRs 1.06–1.25), highest for neurological disease, CKD, stroke, and anemia. Risk rose within 3 years of comorbidity onset, then stabilized, consistent across age, sex, and residential status. Associations persisted across GWTG strata. SGLT2i therapy attenuated mortality risk for most comorbidities, though residual risk remained for stroke, CKD, chronic lung, and neurological disease.

**Conclusions:**

In HFpEF, comorbidities are common and associated with higher mortality, especially within the first 3 years. Prognostic effects persisted across GWTG strata, while SGLT2i therapy attenuated these risks, highlighting the importance of early detection and optimized therapy.

## Abbreviations and acronyms

ACEi/ARBs/ARNi: Angiotensin-Converting Enzyme Inhibitor/Angiotensin II Receptor Blocker/Angiotensin II Receptor Blocker-Neprilysin Inhibitor
CAD: coronary artery disease
CABG: coronary artery bypass grafting
CC: comorbid condition
CI: confidence interval
CKD: chronic kidney disease
CLC: chronic lung condition
COPD: chronic obstructive pulmonary disease
DM: diabetes mellitus
GI: gastrointestinal
GWTG: get guideline
HER: electronic health records
HFpEF: heart failure with preserved ejection fraction
HR: hazard ratio
ICD: implantable cardioverter-defibrillator
LVAD: left ventricular assistive device
MRA: mineralocorticoid receptor antagonist
PCI: percutaneous coronary intervention
STROBE: Strengthening the Reporting of Observational Studies in Epidemiology
VIF: rinse inflation
VHD: valvular heart disease

## 1 Introduction

Heart failure with preserved ejection fraction (HFpEF) is an increasingly important global public health concern, accounting for nearly half of all heart failure (HF) cases worldwide [1]. HFpEF is characterized by a complex interplay of cardiovascular and non-cardiovascular comorbid conditions (CCs), which substantially contribute to its clinical burden, pose challenges in management, affect the prognosis, and increase the healthcare cost [[2], [3], [4]]. These CCs are thought to play a key role in driving structural and functional cardiac changes that lead to the onset, progression, and exacerbation of HFpEF [1,5,6].

Most published studies treated CCs as binary variables (present or absent) when assessing their association with mortality. However, this approach overlooks the duration since diagnosis, or the length of time a patient has lived with a given condition, which may significantly influence the accuracy of mortality risk estimates. Few studies have directly evaluated the association between age at CC diagnosis and mortality; results suggest that earlier diagnosis is associated with higher cumulative mortality [[7], [8], [9], [10]]. Although age at CC diagnosis served as a surrogate marker of disease duration, it may also reflect the cumulative burden of the index condition, such as HF, and co-existing CCs over time, which could independently influence mortality. Accordingly, there is a need to consider comorbidity duration for risk stratification of CCs. Therefore, a significant gap remains in the literature regarding studies that directly investigate how the duration of time a patient lived with CC affects mortality. This gap is particularly critical given that HFpEF represents a spectrum of heterogeneous patient phenotypes with distinct CC clusters [11], and CC prevalence varies by demographic and geographic groups [12]. Importantly, limited research has examined how comorbidity patterns, including their respective preadmission durations, vary across patient subgroups based on age, sex, or residential status, or how these differences influence long-term mortality after HF hospitalization.

This study analyzed a large, retrospectively collected dataset from electronic health records (EHR) of patients hospitalized with HFpEF to address key gaps in existing knowledge. Specifically, we aimed to: (1) assess the prevalence of common CCs at admission and to determine how long patients lived with them before admission (2) determine mortality risks associated with specific CCs; (3) evaluate whether more prolonged duration of CCs was associated with higher mortality; and (4) assess how these associations vary across demographic and geographic subgroups.

## 2 Methodology

### 2.1 Ethics

This study was approved by the Institutional Review Board, which granted a waiver of informed consent due to the retrospective nature of data collection from existing Electronic Health Records (EHR) without direct patient interaction. The research was conducted in accordance with the Strengthening the Reporting of Observational Studies in Epidemiology (STROBE) guidelines [13].

### 2.2 Data source

We abstracted EHR data from patients hospitalized with a primary discharge diagnosis of HFpEF across 4 academic and 13 community hospitals in Arizona, Florida, Minnesota, and Wisconsin over 13 years (January 1, 2010, to December 31, 2022), with follow-up until July 4, 2024. Baseline patient characteristics were captured at admission, while hospital-level characteristics, including the use of guideline-focused medical therapy (GFMT) and discharge destination, were assessed at discharge.

### 2.3 Study design and participants

This is a multicenter longitudinal retrospective cohort study. Inclusion criteria consisted of consecutive adult patients aged >18 years admitted to a participating hospital with a primary diagnosis of heart failure with preserved ejection fraction (HFpEF), defined as a left ventricular ejection fraction (LVEF) ≥50 % or, if LVEF was unavailable, a diagnosis of diastolic heart failure. To avoid duplication, only the first (index) hospitalization for each patient during the study period was analyzed. Exclusion criteria consisted of patients with LVEF <50 %, those who were pregnant, incarcerated, receiving hospice care at the time of admission, or who left the hospital against medical advice. In addition, patients who did not provide written consent through the institution's generic authorization form to allow the use of their EHR data for research were excluded. For patients with available echocardiographic data (obtained during hospitalization or within one year before admission), we applied the guideline-recommended left ventricular ejection fraction (LVEF) threshold of ≥50 % to confirm HFpEF diagnosis [14]. Supplement Fig. 1 illustrates a STROBE flow diagram outlining patient selection.

### 2.4 Risk stratification, heart failure severity

Patients can be risk stratified for in-hospital mortality using the Get With The Guidelines heart failure (GWTG-HF) risk score by calculating a composite score based on readily available clinical variables at admission [15]. The GWTG-HF risk score incorporates age, systolic blood pressure, blood urea nitrogen, heart rate, serum sodium, and the presence of chronic obstructive pulmonary disease. Each variable is assigned a weighted value, and the total score corresponds to a predicted probability of in-hospital mortality. Higher scores indicate greater risk.

### 2.5 Comorbid conditions

The selection of CCs was informed by: (1) prior research from our group [16,17] systematic literature review [18], (3) U.S. Department of Health and Human Services specified CCs [19], and (4) expert consensus. CCs were measured individually and by organ system-based groupings. Supplement Table 1 provides complete documentation of all measured CCs, including their descriptions and corresponding ICD-10-CM codes.

**Individual CC:** included: hypertension, coronary artery disease (CAD), atrial fibrillation/flutter, valvular heart disease, stroke (ischemic/hemorrhagic), diabetes mellitus (types 1 and 2), hyperlipidemia, chronic kidney disease ([CKD], eGFR <60 mL/min/1.73 m^2^), anemia (including nutritional, chronic disease, and hematologic causes), and cancer (hematologic and solid malignancies).

**Organ System-Based CCs:** included chronic lung condition ([CLC], e.g., chronic obstructive pulmonary disease [COPD], bronchiectasis, cystic fibrosis, pulmonary sarcoidosis, asthma, and interstitial lung disease), neurological conditions (such as dementia, Parkinson's disease, multiple sclerosis, epilepsy, and others), gastrointestinal disorders ([GI], such as chronic liver disease, chronic pancreatitis, and inflammatory bowel disease, and others), and rheumatological conditions (e.g., osteoarthritis, gout, rheumatoid arthritis and others).

**Assessment of preadmission CC duration:** CCs were identified from the EHR review, with diagnoses dating back to January 1, 1994. Using the earliest clinician-documented diagnosis for each CC, we calculated preadmission duration and classified CCs into four temporal time-based groups. Based on this timeline, comorbidities were classified into four time-based groups: new (<0.5 years), early (0.5-<3 years), intermediate (3–6 years), and long-term (>6 years).

### 2.6 Objectives

This study had four main objectives: (1) classify admission CCs into four temporal groups (<0.5, 0.5-<3, 3-<6, and ≥6 years) using EHR diagnosis dates; (2) examine associations between measured CCs and long-term mortality; (3) evaluate mortality risk by increasing comorbidity duration; and (4) assess effect modification by age, sex, and rurality.

### 2.7 Mortality ascertainment

Mortality data were extracted from the Mayo Clinic EHR system, which integrates real-time updates from clinical records (hospital and primary care settings), obituaries, death notices, and state death registries as previously described [20].

### 2.8 Follow-up

Patients were followed from the date of their index hospitalization (January 1, 2010) through the study censoring date (July 4, 2024). This design ensured that each patient had at least 18 months of follow-up, with some observed for more than 14 years, reflecting the overall study duration spanning January 2010 to July 2024. We employed a staggered enrollment design [21] to include both early and contemporary patient cohorts. This approach resulted in varying follow-up durations, with earlier enrollees having more extended observation periods and later recruits having shorter follow-up windows, all relative to the study's censoring date. We quantified follow-up time using the reverse Kaplan-Meier method [22], with median follow-up defined as the time at which 50 % of the cohort reached the study's primary endpoint (i.e. death).

### 2.9 Outcome measure

The primary outcome was all-cause mortality, assessed over a median follow-up period estimated using the reverse Kaplan-Meier method.

### 2.10 Subgroups

Subgroup classifications were based on age (<70 vs. ≥70 years), sex (female vs. male), and residential status (rural vs. urban). Separate stratified analyses were conducted within each subgroup. These analyses employed a similar statistical methodology used for the overall cohort to ensure comparability of results across strata.

### 2.11 Statistical analysis

Continuous variables were summarized as means with standard deviations, and categorical variables as percentages. A two-tailed p-value <0.05 was considered statistically significant. Time-to-event analysis for mortality was calculated from admission until death or censoring at the study end date (July 1, 2024). Multivariable Cox regression models were used to estimate adjusted hazard ratios (HRs) and 95 % confidence intervals (CIs) for the association between CCs and mortality. We evaluated the association between incremental comorbidity duration and mortality using restricted cubic splines (RCS) within Cox proportional hazards models. Incremental comorbidity durations were modeled with restricted cubic splines using knots at <0.5, 0.5-<3, 3-<6, and ≥6 years. This flexible modeling approach enabled a more accurate characterization of the nonlinear relationship between comorbidity duration and mortality hazard ratios (HRs) than traditional categorical analyses.

Cox regression models were adjusted to a comprehensive set of independent variables including sociodemographic (age, sex, race, residential status) anthropometric measure (body mass index), prior surgical procedures (coronary angiogram, percutaneous coronary intervention [PCI], pacemaker or implantable cardioverter defibrillator [ICD], left ventricular assist devise [LVAD] implantation, valve repair or replacement, coronary artery bypass surgery [CABG]), vitals (systolic blood pressure [SBP], diastolic blood pressure [DBP], heart rate), laboratory measurements (sodium, blood urea nitrogen (BUN), creatinine levels) disease severity (get with the guideline [GWTG] risk stratification class), admission service (internal medicine, critical care, specialty care), guideline focused medical treatment (Angiotensin-Converting Enzyme Inhibitor/Angiotensin II Receptor Blocker/Angiotensin II Receptor Blocker-Neprilysin Inhibitor [ACEi/ARBs/ARNi], betablocker, mineralocorticoid receptor antagonist [MRA], loop diuretics, anticoagulant [warfarin or non–vitamin K antagonist oral anticoagulant [NOAC]), discharge destination (home, home with home health, rehabilitation, skilled nursing facility, other acute care hospital), 16 predefined CCs (hypertension, coronary artery disease [CAD], atrial fibrillation, valve disease, stroke, hyperlipidemia, diabetes mellitus, obesity, chronic kidney disease [CKD] stage 3 or greater, anemia, chronic lung condition, gastrointestinal or hepatic disease, cancer, rheumatological disease, neurologic disease, psychiatric disease) To assess multicollinearity, we calculated variance inflation factors (VIFs), with a VIF >10 indicating high collinearity. A two-tailed p-value <0.05 was considered statistically significant. Statistical analyses were performed using R version 4.3.1 and SAS Viya (Cary, NC, USA).

We performed a secondary analysis to assess the association between comorbidities and mortality across varying levels of heart failure severity defined by the GWTG scoring system. We also examined whether treatment with sodium–glucose cotransporter-2 inhibitors (SGLT2i), a class of medications originally developed for type 2 diabetes but now recognized for their cardiovascular and renal protective benefits, modified these associations [23,24]. This allowed us to explore the extent to which SGLT2i therapy influenced the relationship between comorbidities and mortality in patients with heart failure.

## 3 Results

### 3.1 Baseline characteristics

A flow diagram of patient selection is provided in Supplement Fig. 1. From an initial screen of 18,773 patients with suspected heart failure, 9256 patients with a primary discharge diagnosis of HFpEF were identified for inclusion, the majority of whom had echocardiographic confirmation of LVEF ≥50 % during hospitalization or within the prior 12 months. Among the initial cohort, 932 patients (5 %) had missing LVEF data. After applying study criteria, 389 (4.2 %) of patients in the final HFpEF cohort had missing LVEF values. In these cases, the physician-documented discharge diagnosis of HFpEF was accepted, and missing LVEF values were imputed using the cohort median for consistency in analyses. The mean age of the study population was 77.8 ± 12.8 (SD) years, with 24 % < 70. Females comprised 54.1 % of the cohort, 4.6 % were identified as non-white, 46.0 % lived with a partner, and 40.2 % resided in rural counties. Between 0.3 % and 26.6 % of patients underwent advanced cardiovascular procedures prior to admission. The median blood urea nitrogen (BUN) was elevated (24 mg/dL), and 73.5 % were classified as GWTG class 2. A total of 5.1 % were admitted to a critical care service. The prescription of GFMT ranged from 5.7 % for sodium-glucose cotransporter-2 inhibitors (SGLT2i) to 42.2 % for diuretics. Finally, the majority were discharged home with self-care (56.5 %), followed by discharge to a skilled nursing facility (23.7 %). Table 1 summarizes the study population's baseline patient- and hospital-level characteristics. Supplement Tables 1, 2, and 3 provide stratified analyses by age, sex, and rurality, highlighting notable differences in patient- and hospital-level characteristics across these subgroups.

**Table 1:** Baseline characteristics of hospitalized HFpEF patients.

Categories	Characteristics	Toal cohort n = 9256
Demographics	Age**,** years, mean ± SD	77.8 ± 12.9
	Age, <70, n= (%)	2221 (24.0)
	Age, ≥70, n= (%)	7035 (76.0)
	Female, n= (%)	5004 (54.1)
	Male, n= (%)	4252 (45.9)
	White, n= (%)	8830 (95.4)
	Non-white, n= (%)	426 (4.6)
Social indicators	Married, n= (%)	4258 (46.0)
	Nonmarried, n= (%)	4998 (54.0)
	Current smoker, n= (%)	966 (10.4)
	Non- or ex-smoker, n= (%)	8290 (89.6)
Anthropometrics	BMI, Kg/m^2^, median (IQR)	30.3 (25.6–36.9)
Pre-admission coronary angiogram PCI, devices, cardiac surgery	Coronary angiogram	2462 (26.6)
	PCI	802 (8.7)
	Pacemaker	773 (7.9)
	ICD	111 (1.2)
	LVAD	21 (0.2)
	Valve repair or replacement	898 (9.7)
	CABG	432 (4.7)
Comorbid conditions	Anemia, n= (%)	1045 (11.3)
	Atrial fibrillation, n= (%)	4630 (50.0)
	CAD, n= (%)	4169 (45.0)
	Cancer, n= (%)	2554 (27.6)
	CKD, n= (%)	2371 (25.6)
	Chronic lung condition, n= (%)	3114 (33.6)
	Diabetes mellitus, n= (%)	4007 (43.3)
	GI and hepatology, n= (%)	1776 (19.2)
	Hyperlipidemia, n= (%)	6251 (67.5)
	Hypertension, n= (%)	6798 (73.4)
	Neurological condition, n= (%)	557 (6.0)
	Obesity, n= (%)	2064 (22.3)
	Psychiatric condition, n= (%)	3381 (36.6)
	Rheumatological condition, n= (%)	3681 (39.8)
	Stroke, n= (%)	1273 (13.8)
	VHD, n= (%)	1727 (18.7)
Vitals	SBP, mmHg, mean ± SD	137 ± 25
	DBP, mmHg, mean ± SD	76 ± 18
	Heart rate, per min, mean ± SD	80 ± 25
Laboratory measures	Sodium, mmol/L, mean ± SD	137.0 ± 5.1
	BUN, mg/dL, Median (IQR)	24.0 (17.0–36.3)
	Creatinine, mg/dL, Median (IQR)	1.1 (0.85–1.40)
Risk stratifications, GWTG	… 1, n= (%)	1255 (13.6)
	… 2, n= (%)	6806 (73.5)
	… 3, n= (%)	1195 (12.9)
Admission service	Internal Medicine, n= (%)	4971 (53.7)
	Critical care, n= (%)	473 (5.1)
	Specialty, n= (%)	3812 (41.2)
Length of stay	Days, median (IQR)	4 (3–6)
Guideline-focused medical treatment	ACEI/ARBs/ARNI, n= (%)	3538 (38.2)
	Beta-blocker, n= (%)	3859 (41.7)
	MRA, n= (%)	2120 (22.9)
	SGLT2 inhibitors, n= (%)	523 (5.7)
	Diuretics, n= (%)	3905 (42.2)
	Anticoagulation, n= (%)	2921 (31.6)
Discharge destination	Home, selfcare	5225 (56.5)
	Home, home health	847 (9.1)
	Rehabilitation	79 (0.8)
	Nursing home	2192 (23.7)
	Other acute care	225 (2.4)
	Hospice	361 (3.9)
	Other destinations	38 (0.4)
	Expired	289 (3.1)

**Abbreviations:** ACE/ARB/ARNI = Angiotensin-Converting Enzyme Inhibitor/Angiotensin Receptor Blocker/Angiotensin Receptor–Neprilysin Inhibitor; BMI = Body Mass Index; BUN = Blood Urea Nitrogen; CABG = Coronary Artery Bypass Grafting; CAD = Coronary Artery Disease; CKD = Chronic Kidney Disease; DBP = Diastolic Blood Pressure; GI = Gastrointestinal; HFpEF = Heart Failure with Preserved Ejection Fraction; ICD = Implantable Cardioverter Defibrillator; IQR = Interquartile Range; LVAD = Left Ventricular Assist Device; MRA = Mineralocorticoid Receptor Antagonist; PCI = Percutaneous Coronary Intervention; SBP = Systolic Blood Pressure; SD = Standard Deviation; SGLT2i = Sodium–Glucose Cotransporter-2 Inhibitor; Valvular HD = Valvular Heart Disease.

### 3.2 Comorbidities

**Fig. 1:**
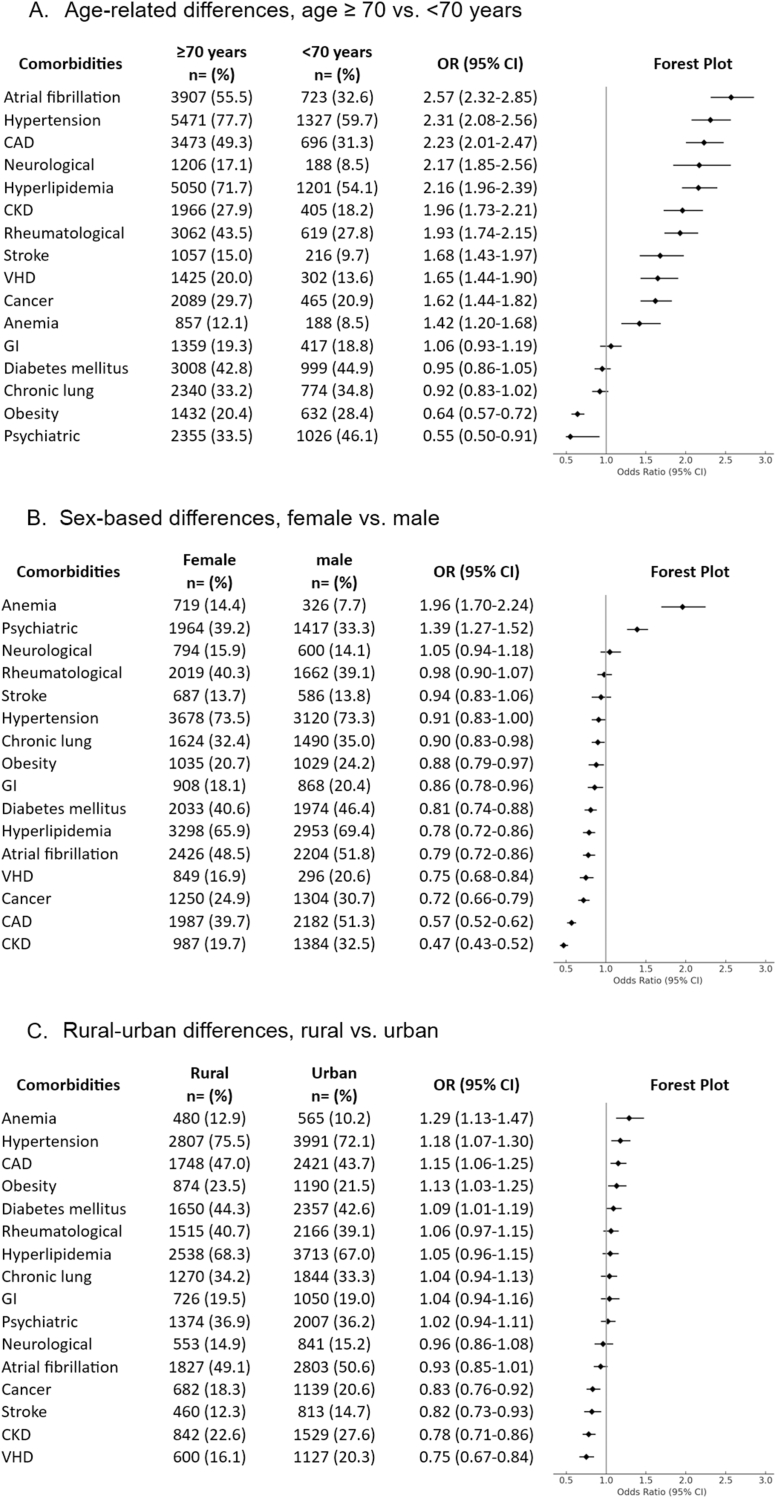
Prevalence of Comorbidities by Age, Sex, and Rural vs. Urban Residence. 1A illustrates adjusted odds ratios (ORs) and 95 % confidence intervals (CI) for differences in the prevalence of measured 16 comorbidities between patients aged <70 and ≥ 70 years, shown by decreasing order of prevalence. 1B illustrates ORs and 95 % CIs for differences in the prevalence of 16 measured comorbidities between patients female and male patients, shown by decreasing order of prevalence. 1C illustrates ORs and 95 % CIs for differences in the prevalence of 16 measured comorbidities between patients from rural counties and those from urban counties, shown by decreasing order of prevalence. **Abbreviations:** CAD, coronary artery disease; CKD, chronic kidney disease, GI, gastrointestinal and hepatic condition; VHD, valvular heart disease.

A total of 656 ICD-10-CM diagnostic codes, corresponding to 71 distinct clinical diagnoses, were systematically aggregated and organized into 14 individual CCs and 4 system-based condition categories to enable structured analysis of comorbidity burden and its association with clinical outcomes in the study cohort (See Supplement Table 1). Table 1 presents the prevalence of 16 CCs among HFpEF patients, with the 10 most frequent being hypertension (73.4 %), hyperlipidemia (67.5 %), atrial fibrillation (50.0 %), diabetes mellitus (43.3 %), rheumatologic conditions (39.8 %), psychiatric conditions (36.5 %), CLC (33.6 %), cancer (27.6 %), CKD (25.6 %), and obesity (22.3 %). Fig. 1 illustrates the distribution of CCs stratified by age (Panel A), sex (Panel B), and urban-rural status (Panel C), expressed as adjusted odds ratios (ORs) with 95 % confidence intervals (CIs), controlling for age, sex, and race. In patients aged ≥70 years, obesity and psychiatric conditions were lower, DM, CLC, and GI/hepatologic conditions were comparable to those <70 years. Other comorbidities were 1.42–2.57 times more frequent, with atrial fibrillation, hypertension, CAD, neurological disorders, and hyperlipidemia being the most prevalent. Female patients had a significantly lower prevalence of CKD, CAD, cancer, VHD, atrial fibrillation, hyperlipidemia, diabetes, GI/hepatologic conditions, and obesity, with comparable rates of hypertension, stroke, and rheumatologic conditions compared to male patients. Anemia and psychiatric conditions were more common in females. Anemia and cardiometabolic conditions were more common in rural patients, while cancer, stroke, CKD, and VHD were less prevalent. Other CCs showed similar prevalence across patients from rural and urban counties.

### 3.3 Multicollinearity

Supplement Figure 2 presents a correlation heatmap with associated VIF values (range: 1.02–1.30), demonstrating the absence of concerning multicollinearity (all VIFs <10) and supporting the inclusion of all CCs in multivariable modeling.

#### 3.3.1 Preadmission comorbidity exposure length

**Fig. 2:**
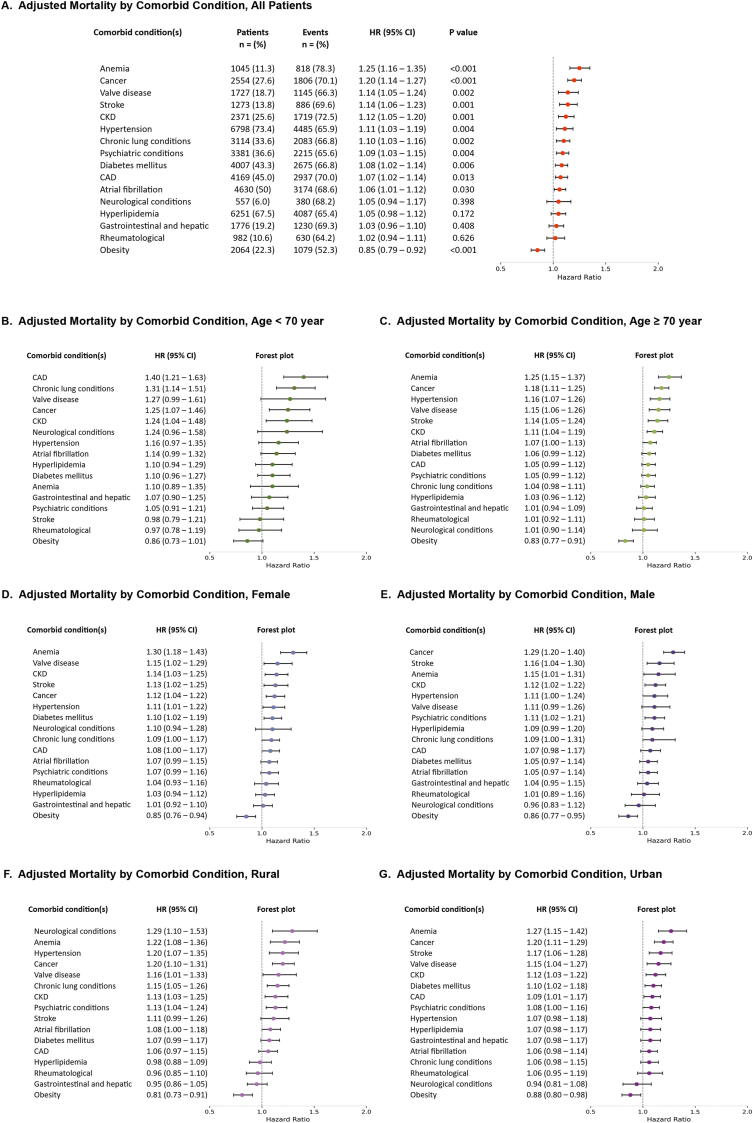
Adjusted Mortality by Comorbid Condition(s) 2A illustrates adjusted hazard ratios (HRs) with 95 % confidence intervals (CI) for all-cause mortality associated with comorbid conditions in the entire cohort, arranged by decreasing HRs. 2B illustrates adjusted HRs with 95 % CIs for all-cause mortality associated with comorbid conditions in patients aged <70 years, arranged by decreasing HRs. 2C illustrates adjusted HRs with 95 % CIs for all-cause mortality associated with comorbid conditions in patients aged ≥70 years, arranged by decreasing HRs. 2D illustrates adjusted HRs with 95 % CIs for all-cause mortality associated with comorbid conditions in female patients, arranged by decreasing HRs. 2E illustrates adjusted HRs with 95 % CIs for all-cause mortality associated with comorbid conditions in male patients, arranged by decreasing HRs. 2F illustrates adjusted HRs with 95 % CIs for all-cause mortality associated with comorbid conditions in patients from rural counties, arranged by decreasing HRs. 2G illustrates adjusted HRs with 95 % CIs for all-cause mortality associated with comorbid conditions in patients from urban counties, arranged by decreasing HRs. **Abbreviations:** CAD, coronary artery disease; CKD, chronic kidney disease.

The median duration of comorbidity exposure varied widely, from over 5 years for diabetes (quartile 3 [Q3] to quartile [Q1], 1.4–8.5 years) to just 0.5 years for obesity (Q3 – Q1, 0.0–1.9 years). As shown in Fig. 2, conditions were stratified by diagnosis duration: <0.5, 0.5-<3.0, 3.0-<6.0, and ≥6 years. Notably, >50 % of CCs were diagnosed within 3 years preceding admission.

### 3.4 Mortality

The median time to death was 2.7 year (IQR 2.6–2.8), overall mortality reached 64 %. Mortality rates differed significantly by age (≥70 years: 70.2 % vs. <70: 44.7 %) and residence (rural: 68.3 % vs. urban: 61.3 %) but were similar between sexes (female: 64.6 % vs. male: 63.5 %).

Most comorbidities were associated with increased mortality with increased risk ranging from 6.0 to 25.1 %. Hyperlipidemia (HR 1.05, 95 % CI 0.98–1.12), gastrointestinal conditions (HR 1.03, 0.96–1.10), rheumatologic disorders (HR 1.02, 0.94–1.11), and neurological conditions (HR 1.05, 0.94–1.17; all p > 0.05) did not have a significant association with mortality. Notably, obesity was associated with a 15.2 % lower mortality risk (HR 0.85; 95 % CI, 0.79–0.92; P = 0.006).

**Fig. 3:**
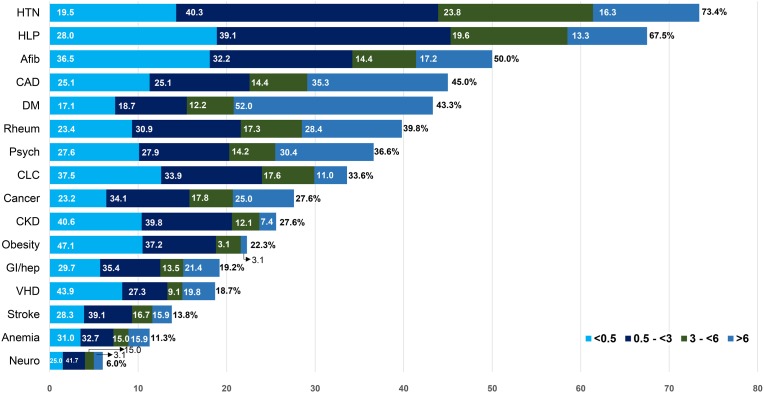
Prevalence and Exposure Duration of Comorbidities in the Entire Cohort. The figure illustrates the percentage of patients with each comorbidity, stratified by four exposure duration categories: <0.5 years, 0.5 to <3 years, 3 to <6 years, and ≥6 years. Comorbidities are listed in descending order of overall prevalence. **Abbreviations:** Afib, atrial fibrillation/flutter; CAD, coronary artery disease; CKD, chronic kidney disease; CLD, chronic lung condition; DM, diabetes mellitus; HLP, hyperlipidemia; HTN, hypertension; GI/hep, gastrointestinal and hepatic condition; Neuro, non-stroke neurological condition; Psych, psychiatric condition; Rheum, rheumatological condition; VHD, valvular heart disease.

Fig. 3 displays the association with mortality stratified by age, sex, and residence type. The strength of association between specific CC and mortality varied by age, sex, and rural vs. urban residence. In patients aged <70 years, CAD (HR, 1.40; 95 % CI, 1.21–1.63) and CLC (HR, 1.31; 95 % CI, 1.14–1.51) were significantly associated with increased mortality, whereas in those aged ≥70 years, anemia (HR, 1.25; 95 % CI, 1.15–1.37), hypertension (HR, 1.16; 95 % CI, 1.07–1.26), VHD (HR, 1.15; 95 % CI 1.06–1.26), and stroke (HR, 1.14; 95 % CI, 1.05–1.24) were significant. Both cancer and CKD were associated with significantly higher mortality across both age groups. Anemia, stroke, cancer, CKD, and hypertension were associated with higher mortality in both sexes. VHD and DM were significant mortality predictors in females, whereas psychiatric condition was associated with greater mortality in male. Anemia, cancer, VHD, and CKD were all associated with higher mortality in patients from both rural and urban counties. In patients from rural residence, neurological conditions (HR 1.29; 95 % CI 1.10–1.53), hypertension (HR 1.20; 95 % CI 1.05–1.24), and CLC (HR 1.15; 95 % CI 1.05–1.26) predicted mortality. In patients from urban counties, diabetes (HR 1.10; 95 % CI 1.02–1.18) and CAD (HR 1.09; 95 % CI 1.01–1.17) were significant. Obesity was consistently associated with a lower mortality in all groups except those <70 years.

### 3.5 Comorbidity duration and mortality

**Fig. 4:**
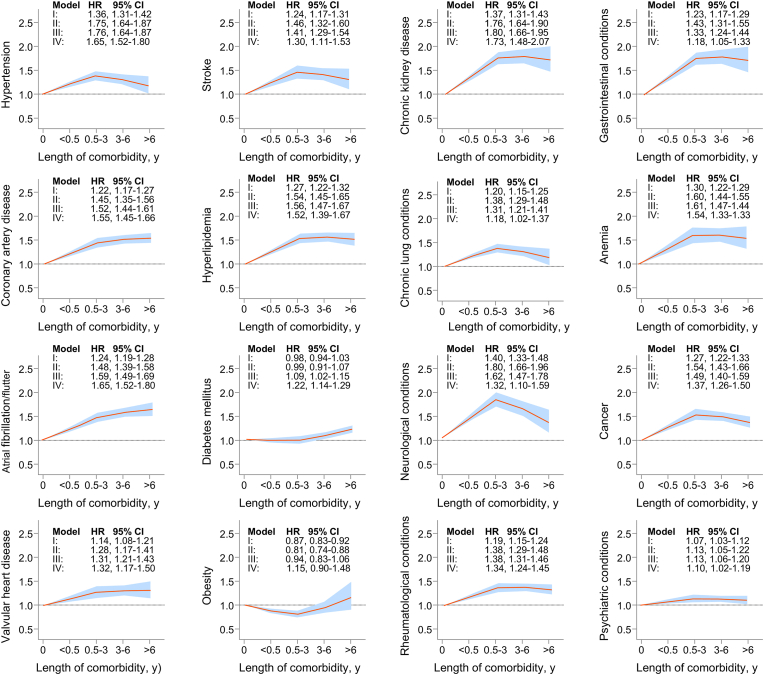
Modeling the Nonlinear Association Between length of Exposure to Comorbidities and Mortality Risk Using Restricted Cubic Spline Within a Cox Proportional Hazards Model.**:** Adjusted hazard ratios (HRs) and 95 % confidence intervals (CIs) for all-cause mortality are shown across the continuous range of comorbidity duration, modeled using restricted cubic splines (RCS) within a multivariable adjusted Cox proportional hazards model. Comorbidity duration was categorized into four levels: <0.5 years, 0.5–<3 years, 3–<6 years, and ≥6 years. RCS functioned with four knots placed at <0.5 years, 0.5–<3 years, 3–<6 years, and ≥6 years. The HR of 1.0 represents the reference point. The solid line represents the estimated mean mortality outcome, and shaded areas correspond to 95 % CI. Separate model was fitted for each measured comorbidity and 4 levels of exposure duration. The model was adjusted both patient- and hospital.

Except for diabetes and obesity, HR for mortality increased from <0.5 to 0.5–3 years of comorbidity duration, then either plateaued, showed a non-significant rise, or declined between 3 and <6 years and beyond. This pattern is illustrated in Fig. 4 using restricted cubic spline analysis with four knots at <0.5 years, 0.5–<3 years, 3 – <6 years, and >6 years of comorbidity duration. For diabetes, a significant association with increased mortality emerged only after 3 (HR, 1.09; 95 % CI 1.02–1.15) or more (HR, 1.22; 95 % CI 1.14–1.29) years of exposure. In contrast, newly diagnosed or early-stage diabetes mellitus was not associated with increased mortality. Obesity diagnosed within <0.5 years (HR 0.87; 95 % CI, 0.83–0.92) or between 0.5 and <3 years before admission (HR 0.81; 95 % CI, 0.74–0.88) was associated with a lower mortality risk. In contrast, obesity that had been present for ≥3 years prior to admission showed no significant association with mortality. These association patterns were largely consistent across subgroups of age, sex, and rural or urban residence (Supplement Figs. 3A,3B, 4A, 4B, 5A, and 5B).

### 3.6 Secondary analysis

As illustrated in the Supplementary Fig. 6, the association between comorbidities and mortality persisted across all levels of heart failure severity (GWTG 1–3). The strength of these associations, however, varied: certain comorbidities such as chronic kidney disease, stroke, neurological disorders, and anemia consistently associated with a higher risk of death, whereas obesity demonstrated a neutral or even inverse association with mortality. Notably, neurological conditions emerged as particularly high-risk predictors in patients with GWTG 1–2, but their effect diminished in those with GWTG scores ≥3, suggesting that baseline severity of heart failure may attenuate the prognostic impact of neurological comorbidity.

**Fig. 5:**
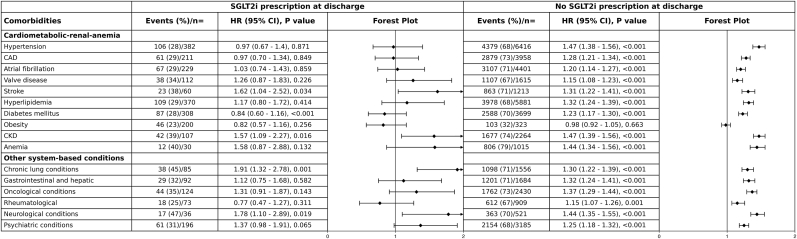
Association of Comorbidities With Mortality at Discharge by SGLT2i Prescription. Caption: Forest plots display hazard ratios (HRs) with 95 % confidence intervals (CIs) for the association between comorbidities and mortality, stratified by discharge prescription of sodium–glucose cotransporter-2 inhibitors (SGLT2i). In the no-SGLT2i group, nearly all comorbidities, including hypertension, CAD, atrial fibrillation, stroke, diabetes, CKD, anemia, chronic lung, oncological, neurological, and psychiatric conditions, were consistently associated with significantly higher mortality (HRs 1.15–1.47; all p < 0.001), with the exception of obesity. In the SGLT2i group, the mortality risk was generally attenuated and did not reach statistical significance for many comorbidities, although risks remained elevated for stroke, CKD, chronic lung, and neurological conditions. Event counts, proportions, HRs, and p-values are presented in the table. Black diamonds indicate point estimates, with horizontal lines representing 95 % CIs.

As shown in the primary Fig. 5, comparisons between patients discharged with and without sodium–glucose cotransporter-2 inhibitor (SGLT2i) prescriptions underscore a potential protective effect of this therapy across a broad spectrum of comorbidities. In the no-SGLT2i group, nearly all comorbidities—including hypertension, coronary artery disease, atrial fibrillation, stroke, diabetes, chronic kidney disease, anemia, chronic lung disease, oncological, neurological, and psychiatric conditions—were consistently and significantly associated with higher mortality, with hazard ratios ranging from 1.15 to 1.47 (all p < 0.001). The only exception was obesity, which did not show a significant association (HR 0.98, p = 0.663). In contrast, in the SGLT2i-treated group, overall mortality risk was attenuated, and for many comorbidities the associations were weaker and did not reach statistical significance, highlighting the potential mitigating role of SGLT2i therapy.

## 4 Discussion

This study represents the first systematic evaluation of how the duration of CCs, categorized into four preadmission intervals, relates to long-term mortality following hospitalization for HF, with further analysis by age, sex, and rurality. A novel finding was that the association between CC duration and mortality was neither linear nor dose-dependent. Instead, mortality risk increased early and stabilized within three years for most CCs, except for diabetes and obesity. This implies a threshold effect where prolonged exposure does not heighten risk beyond a certain point. Additionally, the study revealed a high comorbidity burden in hospitalized HFpEF patients, surpassing rates seen in earlier general hospital [25] and community-based populations [26,27]. The predominant CCs varied by age, sex, and rural-urban residence, with most conferring a modest (6.0–25.1 %) but statistically significant increase in mortality risk. Notably, anemia, cancer, VHD, stroke, and hypertension showed significant associations with mortality in the overall cohort. At the same time, subgroup-specific risks were driven by CAD (in patients <70 years), anemia (in women and older adults), cancer (in men), neurological conditions (in rural residents), and stroke (in urban residents). The findings also reinforced the obesity paradox, demonstrating that obesity was consistently linked to lower mortality across all subgroups except those <70 years. Additionally, secondary analyses showed that comorbidities were associated with increased mortality across all levels of heart failure severity (GWTG 1 to 3). Prescription of SGLT2i at discharge appeared to attenuate these risks, suggesting it may represent a potentially beneficial strategy to mitigate the effect of comorbidities in patients with HFpEF.

### 4.1 Clinical context

Existing literature documented an increasing burden of cardiovascular and non-cardiovascular CCs among HF patients [4,18,28,29], generally consistent with our results, but higher than those reported in earlier studies [30]. Differences in comorbidity prevalence between contemporary and earlier studies may reflect evolving patient populations, improved EHR utilization, heightened clinical recognition, and potential reporting biases. Prior research has documented an association between specific comorbidities and increased mortality in HF, potentially influenced by differences in patient demographics and geographic setting, but the strength of these associations has varied [29,30].

Prior studies investigating the effects of disease duration have relied on cumulative mortality outcomes without accounting for variations in exposure time. Most investigated single comorbidities (e.g., hypertension or diabetes) rather than assessing multiple conditions concurrently. The literature suggests that mortality risk typically follows a nonlinear pattern, with an initial rise, followed by stabilization or decline over time, though cumulative risk remains clinically significant. Notably, large European and Asian population studies observed this pattern with hypertension, where risk plateaued or decreased after an initial rise [8,10]. Age modifies this relationship, with risk stabilization occurring after approximately 6 years in patients ≥75 years [9]. Similarly, diabetes duration demonstrates cumulative mortality effects based on a meta-analysis, though with significant interstudy heterogeneity and age-dependent complications. [31]. Most complications related to diabetes duration were influenced by age. [32,33]. CKD exhibits comparable dynamics, with mortality risk sharply rising in the first 6 years post-diagnosis before stabilizing [34]. Our study extends these observations to hospitalized HFpEF patients by systematically evaluating the duration of exposure to multiple prevalent comorbidities within a unified cohort and supporting a nonlinear risk trajectory. We extended the observed benefits of SGLT2i therapy beyond its well-established cardiovascular and renal protective effects in patients with heart failure. Our findings suggest that SGLT2i may also exert an attenuating influence on the adverse effect of a broad range of comorbidities and their association with long-term mortality. These results are consistent with a recent report indicating that SGLT2i therapy may mitigate mortality risks associated with a wide spectrum of hospital-acquired conditions [35], further supporting its potential as a multifaceted therapeutic intervention in heart failure care.

Inflammation may be a central driver in the pathogenesis and progression of HFpEF and its common comorbidities, including obesity, diabetes, CKD, rheumatological conditions, and cancer. In HFpEF, systemic proinflammatory states induced by obesity, diabetes, and CKD contribute to coronary microvascular endothelial inflammation, reduced nitric oxide bioavailability, and subsequent myocardial stiffening and fibrosis, a paradigm well supported by mechanistic and clinical data [[1], [2], [3], [4]]. The American College of Cardiology emphasizes obesity as a major risk factor for HFpEF through chronic inflammation and metabolic dysregulation [5], with adipose tissue expansion, macrophage infiltration, and cytokine release (e.g., TNF-α, IL-6, MCP-1) driving insulin resistance, diabetes, and cardiovascular and renal complications [6,7]. In diabetes, persistent activation of innate immune pathways accelerates vascular and microvascular damage [8,9], while in cancer, chronic inflammatory signaling promotes mutagenesis, angiogenesis, and tumor progression [10,11]. Similarly, in CKD, low-grade inflammation fosters renal fibrosis, hypertension, maladaptive repair, and heightened cardiovascular risk through dysregulated immune responses and tertiary lymphoid structure formation [12,13].

### 4.2 Clinical implications

Our findings suggest that early intervention, particularly within the first three years of CC diagnosis, is critical for potentially improving long-term survival in HFpEF patients. The substantial variation in dominant CCs and associated mortality risks across age, sex, and rural-urban residence underscores the importance of personalized, demographically, and geographically tailored comorbidity management strategies. Additionally, the study reaffirms the obesity paradox in HFpEF, with obesity consistently demonstrating an inverse association with mortality, indicating further personalization of CC management. Finally, the use of SGLT2i therapy, which in our analysis appeared to attenuate the adverse effect of comorbidities on mortality, offers additional rationale for prioritizing its initiation following a heart failure hospitalization. Beyond its established benefits in improving cardiovascular and renal outcomes, our findings suggest that SGLT2i may also help mitigate the prognostic burden of common comorbidities, reinforcing its role as a key component of guideline-directed medical therapy [23,24].

### 4.3 Strengths and limitations

This study's primary strength lies in its comprehensive evaluation of multiple CCs and their preadmission durations within a large, well-characterized cohort of hospitalized HFpEF patients, with follow-up extending up to 14 years. Unlike prior studies that treated comorbidities as binary (present/absent) variables, we quantified exposure duration, offering a more nuanced assessment of their mortality impact. Additionally, our stratified analyses by age, sex, and residence revealed subgroup-specific mortality patterns, enhancing clinical applicability for personalized care.

The study has significant limitations. The retrospective design carries inherent risks of unmeasured confounding. Comorbidity durations were derived from physician-documented diagnoses in EHR, which may not precisely reflect actual disease onset. However, this method remains superior to self-reported data prone to recall bias. [[36], [37], [38]] We specifically acknowledge the emerging classifications of obesity phenotypes, including metabolically unhealthy, metabolically healthy, and metabolically neutral obesity. These distinctions represent important areas of ongoing investigation, as they may carry different prognostic implications. However, the data available in our cohort were insufficient to reliably differentiate between these subtypes, and therefore we were unable to incorporate them into the present analysis. Generalizability may be limited to hospitalized HFpEF populations, as the study did not include outpatient or other HF phenotypes. Furthermore, we could not account for temporal changes in disease severity or treatment regimens, which may influence long-term outcomes. Despite these constraints, our findings provide valuable evidence for understanding comorbidity-associated risks in HFpEF.

### 4.4 Conclusions

This study provides the first comprehensive evaluation of comorbidity duration and long-term mortality in hospitalized HFpEF patients. We found that most comorbidities were highly prevalent and were associated with modest but significant mortality risk, rising over the first three years before stabilizing. Risk patterns were consistent across age, sex, and rurality, underscoring the need for personalized care strategies. Importantly, SGLT2i therapy appeared to attenuate the adverse impact of several comorbidities, extending its benefits beyond cardiovascular and renal protection. These findings highlight the importance of early comorbidity detection, timely intervention, and incorporation of SGLT2i into guideline-directed management to improve HFpEF outcomes.

## CRediT authorship contribution statement

**Mohammed Yousufuddin:** Writing – review & editing, Writing – original draft, Visualization, Validation, Supervision, Software, Resources, Project administration, Methodology, Investigation, Formal analysis, Data curation, Conceptualization. **Zeliang Ma:** Writing – review & editing, Writing – original draft, Visualization, Validation, Supervision, Software, Resources, Methodology, Investigation, Formal analysis, Conceptualization. **Ebrahim Barkoudah:** Writing – review & editing, Writing – original draft, Visualization, Validation, Supervision, Resources, Methodology, Investigation, Conceptualization. **Muhammad Waqas Tahir:** Writing – review & editing, Writing – original draft, Visualization, Validation, Software, Methodology, Investigation. **Ali Yazdanyar:** Writing – review & editing, Writing – original draft, Visualization, Validation, Supervision, Resources, Methodology, Investigation, Conceptualization. **Rani Chikkanna:** Writing – review & editing, Writing – original draft, Visualization, Supervision, Resources, Methodology, Investigation. **Khalid Benkhadra:** Writing – review & editing, Writing – original draft, Visualization, Validation, Supervision, Methodology, Investigation. **Sumit Bhagra:** Writing – review & editing, Writing – original draft, Visualization, Validation, Software, Resources, Methodology, Investigation, Conceptualization. **Gregg C. Fonarow:** Writing – review & editing, Writing – original draft, Visualization, Validation, Supervision, Methodology, Conceptualization. **Mohamad H. Yamani:** Writing – review & editing, Writing – original draft, Visualization, Validation, Supervision, Resources, Project administration, Methodology, Investigation, Conceptualization.

## Ethical consideration

The study was approved by the Mayo Clinic Institutional Review Board, which granted a waiver of informed consent due to the retrospective nature of data collection from existing Electronic Health Records without direct patient interaction (ID # 22–013354).

## Data sharing statement

Deidentified individual participant data (IPD) will be made available upon reasonable request to the corresponding author to reproduce the results of this study, subject to institutional approvals.

## Funding

No funding was received to conduct this study.

## Declaration of competing interest

Dr. Fonarow reports consulting for Abbott, Amgen, AstraZeneca, Bayer, Boehringer Ingelheim, Cytokinetics, Eli Lilly, Johnson & Johnson, Medtronic, Merck, and Pfizer. Dr. Barkoudah reports research support payments from the 10.13039/100000002National Institutes of Health/10.13039/100000050National Heart, Lung, and Blood Institute, and contracts all made to Brigham and Women's Hospital; payments made to Brigham and Women's Hospital for performing clinical endpoints sponsored by various entities including serving on trials committee; payments from Medscape and WebMD (editor-in-chief of JCOM), and Advisory Board fees from Bayer, Gilead and Novartis. There were non-compensated efforts in consulting through OSG, CaptiOX, and volunteer board positions. All outside the submitted work. The other authors have no disclosures to report.

## References

[1] Martin S.S., Aday A.W., Almarzooq Z.I., Anderson C.A.M., Arora P., Avery C.L. (2024). 2024 heart disease and stroke statistics: a report of US and global data from the American heart association. Circulation.

[2] Ather S., Chan W., Bozkurt B., Aguilar D., Ramasubbu K., Zachariah A.A. (2012). Impact of noncardiac comorbidities on morbidity and mortality in a predominantly male population with heart failure and preserved versus reduced ejection fraction. J. Am. Coll. Cardiol..

[3] Chunawala Z.S., Qamar A., Arora S., Pandey A., Fudim M., Vaduganathan M. (2022). Prevalence and prognostic significance of polyvascular disease in patients hospitalized with acute decompensated heart failure: the ARIC study. J. Card. Fail..

[4] Pandey A., Vaduganathan M., Arora S., Qamar A., Mentz R.J., Shah S.J. (2020). Temporal trends in prevalence and prognostic implications of comorbidities among patients with acute decompensated heart failure: the ARIC study community surveillance. Circulation.

[5] Campbell P., Rutten F.H., Lee M.M.Y., Hawkins N.M., Petrie M.C. (2024). Heart failure with preserved ejection fraction: everything the clinician needs to know. Lancet.

[6] Mishra S., Kass D.A. (2021). Cellular and molecular pathobiology of heart failure with preserved ejection fraction. Nat. Rev. Cardiol..

[7] (2023). Life expectancy associated with different ages at diagnosis of type 2 diabetes in high-income countries: 23 million person-years of observation. Lancet Diabetes Endocrinol..

[8] Kim T.H., Yang P.S., Yu H.T., Jang E., Shin H., Kim H.Y. (2019). Effect of hypertension duration and blood pressure level on ischaemic stroke risk in atrial fibrillation: nationwide data covering the entire Korean population. Eur. Heart J..

[9] Wang C., Yuan Y., Zheng M., Pan A., Wang M., Zhao M. (2020). Association of age of onset of hypertension with cardiovascular diseases and mortality. JACC (J. Am. Coll. Cardiol.).

[10] Zheng Y., Gao X., Jia H.Y., Li F.R., Ye H. (2022). Influence of hypertension duration and blood pressure levels on cardiovascular disease and all-cause mortality: a large prospective cohort study. Front. Cardiovasc. Med..

[11] Li M., Lip G.Y.H. (2024). Contemporary heart failure and comorbidity risk management. Lancet Public Health.

[12] Boersma P., Black L.I., Ward B.W. (2020). Prevalence of multiple chronic conditions among US adults, 2018. Prev. Chronic Dis..

[13] von Elm E., Altman D.G., Egger M., Pocock S.J., Gøtzsche P.C., Vandenbroucke J.P. (2007). The strengthening the reporting of observational studies in epidemiology (STROBE) statement: guidelines for reporting observational studies. Ann. Intern. Med..

[14] Yancy C.W., Jessup M., Bozkurt B., Butler J., Casey D.E., Colvin M.M. (2017). 2017 ACC/AHA/HFSA focused update of the 2013 ACCF/AHA guideline for the management of heart failure: a report of the American college of cardiology/american heart association task force on clinical practice guidelines and the heart failure society of America. J. Am. Coll. Cardiol..

[15] Peterson P.N., Rumsfeld J.S., Liang L., Albert N.M., Hernandez A.F., Peterson E.D. (2010).

[16] Yousufuddin M., Bartley A.C., Alsawas M., Sheely H.L., Shultz J., Takahashi P.Y. (2017). Impact of multiple chronic conditions in patients hospitalized with stroke and transient ischemic attack. J. Stroke Cerebrovasc. Dis..

[17] Yousufuddin M., Yamani M.H., Kashani K.B., Zhu Y., Wang Z., Seshadri A. (2023). Characteristics, treatment patterns, and clinical outcomes after heart failure hospitalizations during the COVID-19 pandemic, march to October 2020. Mayo Clin. Proc..

[18] Khan M.S., Samman Tahhan A., Vaduganathan M., Greene S.J., Alrohaibani A., Anker S.D. (2020). Trends in prevalence of comorbidities in heart failure clinical trials. Eur. J. Heart Fail..

[19] Goodman R.A., Posner S.F., Huang E.S., Parekh A.K., Koh H.K. (2013). Defining and measuring chronic conditions: imperatives for research, policy, program, and practice. Prev. Chronic Dis..

[20] Henkel D.M., Redfield M.M., Weston S.A., Gerber Y., Roger V.L. (2008). Death in heart failure: a community perspective. Circ Heart Fail.

[21] King E.C., Doherty M., Corcos D., Stoykov M.E. (2020). Examining recruitment feasibility and related outcomes in adults post-stroke. Pilot Feasibility Stud.

[22] Schemper M., Smith T.L. (1996). A note on quantifying follow-up in studies of failure time. Control. Clin. Trials.

[23] Heidenreich P.A., Bozkurt B., Aguilar D., Allen L.A., Byun J.J., Colvin M.M. (2022). 2022 AHA/ACC/HFSA guideline for the management of heart failure: a report of the American college of cardiology/american heart association joint committee on clinical practice guidelines. J. Am. Coll. Cardiol..

[24] Usman M.S., Bhatt D.L., Hameed I., Anker S.D., Cheng A.Y.Y., Hernandez A.F. (2024). Effect of SGLT2 inhibitors on heart failure outcomes and cardiovascular death across the cardiometabolic disease spectrum: a systematic review and meta-analysis. Lancet Diabetes Endocrinol..

[25] Owens P.L., Liang L., Barrett M.L., Fingar K.R. (2006). Healthcare Cost and Utilization Project (HCUP) Statistical Briefs.

[26] Barnett K., Mercer S.W., Norbury M., Watt G., Wyke S., Guthrie B. (2012). Epidemiology of multimorbidity and implications for health care, research, and medical education: a cross-sectional study. Lancet.

[27] Fortin M., Stewart M., Poitras M.E., Almirall J., Maddocks H. (2012). A systematic review of prevalence studies on multimorbidity: toward a more uniform methodology. Ann. Fam. Med..

[28] Adams K.F., Fonarow G.C., Emerman C.L., LeJemtel T.H., Costanzo M.R., Abraham W.T. (2005). Characteristics and outcomes of patients hospitalized for heart failure in the United States: rationale, design, and preliminary observations from the first 100,000 cases in the acute decompensated heart failure national registry (ADHERE). Am. Heart J..

[29] Mentz R.J., Kelly J.P., von Lueder T.G., Voors A.A., Lam C.S., Cowie M.R. (2014). Noncardiac comorbidities in heart failure with reduced versus preserved ejection fraction. J. Am. Coll. Cardiol..

[30] Braunstein J.B., Anderson G.F., Gerstenblith G., Weller W., Niefeld M., Herbert R. (2003). Noncardiac comorbidity increases preventable hospitalizations and mortality among medicare beneficiaries with chronic heart failure. J. Am. Coll. Cardiol..

[31] Wang X-m, Zhong S-p, Li G-f, Zhuge F-y (2023). Diabetes duration or age at onset and mortality in insulin-dependent diabetics: a systematic review and meta-analysis. Diabetol. Metab. Syndr..

[32] Cigolle C.T., Blaum C.S., Lyu C., Ha J., Kabeto M., Zhong J. (2022). Associations of age at diagnosis and duration of diabetes with morbidity and mortality among older adults. JAMA Netw. Open.

[33] Holman N., Wild S.H., Gregg E.W., Valabhji J., Sattar N., Khunti K. (2022). Comparison of mortality in people with type 1 and type 2 diabetes by age of diagnosis: an incident population-based study in England and Wales. Lancet Diabetes Endocrinol..

[34] Kim K.M., Oh H.J., Choi H.Y., Lee H., Ryu D.R. (2019). Impact of chronic kidney disease on mortality: a nationwide cohort study. Kidney Res Clin Pract.

[35] Yousufuddin M., Yamani M.H., DeSimone D., Barkoudah E., Tahir M.W., Ma Z. (2025). In-Hospital adverse events in heart failure patients: Incidence and association with 90-Day mortality. Joint Comm. J. Qual. Patient Saf..

[36] Chen V. (2006). Recall bias in self-reporting of familial cancer. Lancet Oncol..

[37] Fox K.A.A., Accetta G., Pieper K.S., Bassand J.-P., Camm A.J., Fitzmaurice D.A. (2017). Why are outcomes different for registry patients enrolled prospectively and retrospectively? Insights from the global anticoagulant registry in the FIELD-atrial fibrillation (GARFIELD-AF). European Heart Journal - Quality of Care and Clinical Outcomes.

[38] Gliklich R.E.D.N., MB L. (2014). Registries for Evaluating Patient Outcomes.

