# Supplementary material for: Comorbidity patterns and mortality in HFpEF: A retrospective longitudinal cohort study
Source: Int J Cardiol Cardiovasc Risk Prev. 2025 Oct 10;27:200526. doi: 10.1016/j.ijcrp.2025.200526 (PMC12549775; doi:10.1016/j.ijcrp.2025.200526)
Supplement: Multimedia component 1 [file mmc1.pdf]

Supplement

Comorbidity Patterns and Mortality in HFpEF: A Retrospective Longitudinal Cohort Study

	Page
**Supplement Figure 1**. STROBE flow diagram………………………………………………………………..	2
**Supplement Table 1**. Categories, descriptions, and ICD-10-CM codes for comorbid conditions……………….	3
**Supplement Table 2.** Baseline characteristics of HFpEF patients stratified by age <70 vs. ≥70 years…...........	6
**Supplement Table 3**. Baseline characteristics of HFpEF patients stratified by sex, female vs. male…………...	8
**Supplement Table 4.** Baseline characteristics of HFpEF patients stratified by rural vs. urban residency………	10
**Supplement Figure 2.** Heat Map illustrating correlation matrix……………………………………………...	12
**Supplement Figure 3A.** Adjusted HRs and 95% CI for mortality by comorbidity duration, age < 70 years.....	13
**Supplement Figure 3B.** Adjusted HRs and 95% CI for mortality by comorbidity duration, age ≥ 70 years….	15
**Supplement Figure 4A.** Adjusted HRs and 95% CI for mortality by comorbidity duration, female…………	17
**Supplement Figure 4B.** Adjusted HRs and 95% CI for mortality by comorbidity duration, female…………	19
**Supplement Figure 5A.** Adjusted HRs and 95% CI for mortality by comorbidity duration, rural……………	21
**Supplement Figure 5B.** Adjusted HRs and 95% CI for mortality by comorbidity duration, urban…………..	23
**Supplement Figure 6.** Association between comorbidities and mortality across heart failure severity defined by GWTG score…………………………………………………….	25
**Supplement Table 5.** STROBE Check list………………………………………………………………	26

Supplement Figure 1. STROBE flow diagram illustrating patient selection for the study.

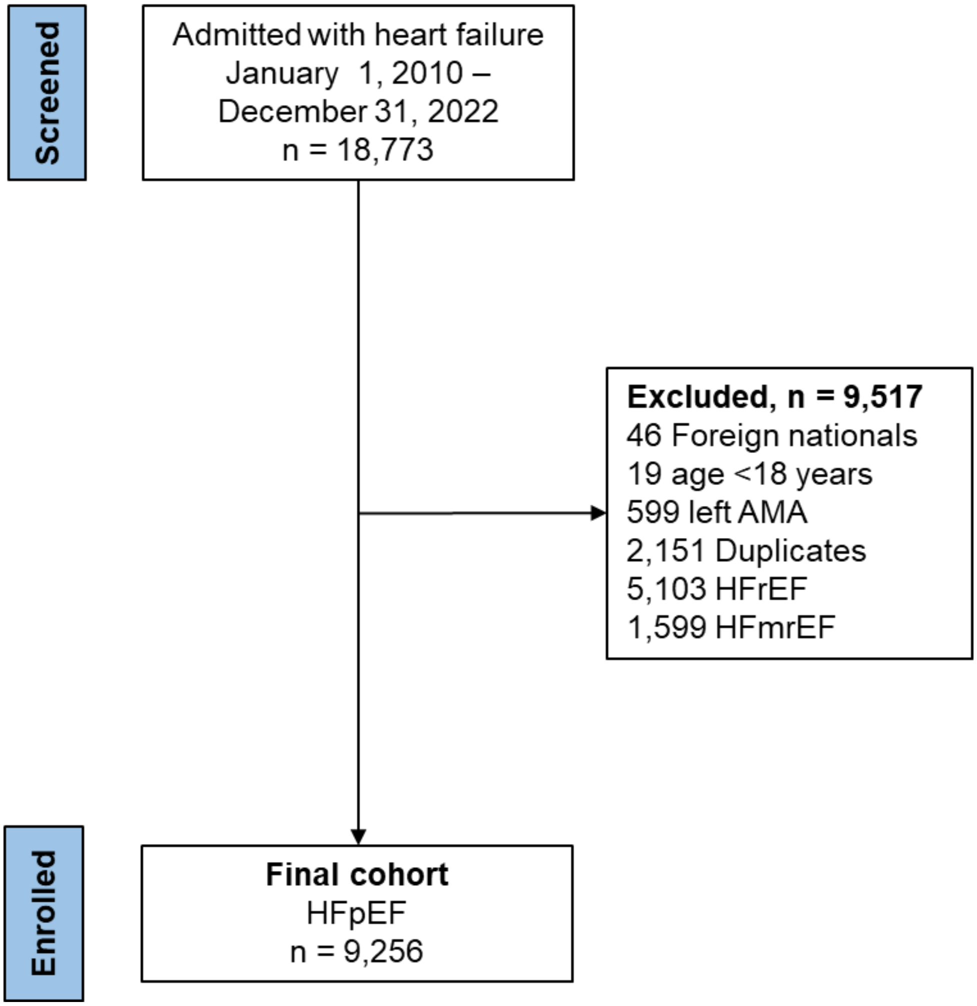

Abbreviations: AMA, against medical advice; HFmrEF, heart failure with mildly reduced ejection fraction; HFpEF, heart failure with preserved ejection fraction; HFrEF, heart failure with reduced ejection fraction; STROBE, Strengthening the Reporting of Observational Studies in Epidemiology.

Supplement Table 1. Categories, descriptions, and ICD-10-CM codes for comorbid conditions

Category	Description of comorbid condition	ICD-10-CM codes
**Cardiovascular**	Hypertension<br>*Primary and secondary hypertension*	I10, I11.9, I15.0, I15.9
	Coronary artery disease<br>Prior acute coronary syndrome, stress-induced myocardial ischemia, prior PCI or CABG	I20.0, I21.4, I21.AI, I25.42, I25.82, I25.110, I25.111, I125.118, I25.119, I25.710, Z95.1, Z95.5, Z98.61, Z98.61
	Atrial fibrillation or flutter<br>Paroxysmal, persistent, and permanent	I48.0, I48.1, I48.11, I48.19, I48.2, I48.20, I48.3, I48.4, I48.91, I48.91
	Stroke<br>Ischemic and hemorrhagic	I61.11, I60.12, I60.31, I60.32, I60.4, I60.51, I60.6, I60.7, I60.8, I60.9, I61.1, I61.2, I61.5, I61.6, I61.8, I61.9, I62.00I63.00, I63.011, I63.012, I63.019, I63.02, I63.22, I63.233, I63.30, I63.031, I63.032, I63.10, I63.311, I63.20, I63.212, I63.219, I63.239, I63.321, I63.232, I63.312, I63.321, I63.322, I63.332, I63.331, I63.333, I63.39, I63.341, I63.342, I63.343, I63.413, I63.419, I63.429, I63.431, I63.432, I63.439, I63.40, I63.511, I63.512, I63.513, I63.521, I63.522, I63.523, I63.532, I63.541, I63.542, I63.543, I63.50, I63.59, I63.8, I63.81, I63.89, I63.9, I69.054, I69.122, I69.151, I69.154, I69.198, I69.222, I69.251, I69.254, I69.328, I69.331, I69.332, I69.331, I69.334, I69.352, I69.354, I69.359, I69.392, I69.398, I69.822, I69.851, I69.852, I69.854, I69.922, I69.928, I69.931, I69.934, I69.939, I69.951, I69.954, I69.959, I69.992, G81.90, G81.91, G81.92, G81.93, G81.94
	Valvular heart disease<br>Moderate to severe aortic, pulmonary, mitral and tricuspid valve disease or prior repair or replacement	I05.0, 105.1, I05.2, I05.8, I05.9, I07.1, I07.2, I07.8, I07.9, I08.0, I08.2, I08.3, I08.8, I08.9 I09.0, I34.0, I34.1, I34.2, I34.9, I35.0, I35.1, I35.2, I35.9, I36.0, I36.1, I36.2, I36.9, I37.0, I37.1, I37.2, I37.9, V43.3, Z95.2, Z95.3, Z95.4, Z95.9
**Chronic lung conditions**	Asthma, COPD, OSA, bronchiectasis, sarcoidosis, pulmonary fibrosis, obesity-hypoventilation, chronic respiratory failure	D86.0, D86.2, G47.33, J44.0, J44.1, J44.9, J45.20, J45.21, J45.22, J45.30, J45.41, J45.42, J45.50, J45.51, J45.52, J45.901, J45.909, J47.0, J47.1, J47.9, J84.10, J96.11
**Neurological conditions**	Dementia, seizures, Parkinson disease, multiple sclerosis, non-traumatic sub-dural hematoma	F02.80, F02.81, F0.90, F0.91, F01.50, F0.51, G20, G30.0, G30.1, G30.9, G31.09, G31.83, G35, G40.301, G40.309, G40.311 G40.319, G40.501, G40.509 G40.901, G40.909, G40.911, G40.319, G40.301, I62.01, I62.03
**Endocrine and**	Hyperlipidemia	E78.00, E78.2, E78.5

**Metabolism**	Diabetes mellitus (Type 1 or Type 2)	E08.9, E08.10, E08.11, E08.21, E08.22, E08.40, E08.42, E08.52, E08.65, E08.649, E08.620, E08.3513, E08.3593, E10.9, E10.10, E10.11, E10.21, E10.22, E10.36, E10.40, E10.40, E10.43, E10.51, E10.52, E10.65, E10.69,E10.319, E10.621, E10.641, E10.649,E10.3513, E10.3593,
	Obesity	E66.01, E66.09, E66.09, E66.2, E66.8, E66.9, Z68.30, Z68.31, Z68.32, Z68.33, Z68.34, Z68.35, Z68.36, Z68.37, Z68.38, Z68.39, Z68.40, Z68.41, Z68.42, Z68.43, Z68.44, Z68.45
**Gastrointestinal and hepatic conditions**	Liver cirrhosis, hemochromatosis, chronic viral hepatitis, Ulcerative colitis, Crohn's disease	B18.0, B18.1, B18.2, E83.110, E83.119, I85.00, I85.01, K29.20, K29.21, K50, K51, K70.0, K70.0, K70.2, K70.9, K70.10, K70.11, K70.30, K70.31 K70.40, K70.41, K72.00, K72.01, K72.90, K72.91, K74.3 K74.5, K74.60, K76.0, K76.7, K76.81, K76.82
**Kidney**	Chronic kidney disease	N18.3, N18.31, N18.32, N18.4, N18.5, N18.6, Z99.2,
**Hematology**	Anemia	D50.0, D52.9, D63.0, D63.1, D63.8, D64.81, D64.9
**Rheumatological conditions**	Osteoarthritis, gout, rheumatoid arthritis, systemic lupus erythematosus, systemic sclerosis, unspecified inflammatory spondylopathy, Antiphospholipid syndrome, CREST syndrome, Ankylosing spondylitis, inflammatory polyarthropathy.	D47.02, D68.61, D68.382, M1A, M05, M06, M10, M16, M17, M18, M31, M34, M45, M46, M48, V43,
**Cancer**	Non-dermatological cancers	C01, C02.1, C02.3, C02.9, C03.9, C06.1, C06.9, C07, C7A.00, C7A.010, C7A.012, C7A.019, C7A.020, C7A.025, C7A.090, C7A.092, C7A.098, C10.9, C11.9, C14.0, C15.4, C15.5. C15.8, C15.9, C16.0, C16.1, C16.2, C16.5, C16.6, C16.9, C17.2, C18.0, C18.1, C18.2, C18.3, C18.4, C18.5, C18.6, C18.7, C18.8, C18.9, C19, C20, C21.0, C21.1, C21.8, C22.0, C22.1, C22.9, C23, C24.0, C24.1, C24.9, C25.0, C25.1, C25.2, C25.4, C25.7, C25.9, C26.1, C26.9, C30.0, C32.1, C32.9, C34.01, C34.02, C34.10, C34.11, C34.12, C34.2, C34.30, C34.31, C34.32, C32.8, C34.80, C34.81, C34.82, C34.90, C34.91, C34.92, C37, C38.3, C41.1, C41.2, C41.4, C41.9, C47.9, C48.0, C48.2, C49.0, C49.21, C49.22, C49.3, C49.4, C49.8, C49.9, C50.012, C50.111, C50.112, C50.119,C50.211, C50.212, C50.312, C50.411, C50.412, C50.512, C50.811, C50.812, C50.819, C50.911, C50.912, C50.919, C51.0, C51.9, C53.0, C53.8, C53.9, C54.1, C55, C56.1, C56.2, C56.3, C56.9, C57.01, C57.7, C57.8, C57.9, C60.9, C61, C62.11, C64.1, C64.2, C64.9, C65.9, C66.1, C66.2, C66.9, C67.0, C67.1, C67.2, C67.4, C67.6, C67.8, C67.9, C68.8, C69.2, C69.21, C69.22, C69.31, C69.32, C70.1,C71.2, C71.3, C71.1, C71.6, C71.8, C71.9, C72.0, C73, C74.91,

		C74.92, C74.99, C75.9, C76.0, C76.1, C76.3, C76.42, C76.52, C76.8, C81.00, C81.02, C81.08, C81.10, C81.12, C81.18, C81.19, C81.90, C82.00, C82.02, C82.03, C82.04, C82.08, C82.09, C82.10, C82.11, C82.13, C82.15, C82.16, C82.17, C82.18, C82.19, C82.20, C82.21, C82.28, C82.29, C82.31, C82.38, C82.39, C82.40, C82.48, C82.49, C82.58, C82.80, C82.88, C82.89, C82.90, C82.93, C82.98, C82.99, C83.00, C83.01, C83.02, C83.03, C83.06, C83.07, C83.09, C83.10, C83.11, C83.12, C83.13, C83.18, C83.19, C83.30, C83.31, C83.32, C83.33, C83.34, C83.36, C83.37, C83.38, C83.39, C83.70, C83.73, C83.78, C83.79, C84.40, C84.48, C84.49, C84.60, C84.70, C84.71, C84.73, C84.74, C84.78, C84.79, C85.20, C85.22, C85.28, C85.29, C85.80, C85.82, C85.83, C85.88, C85.89, C85.90, C85.91, C85.92, C85.93, C85.94, C85.96, C85.98, C85.99, C86.0, C88.0, C88.4, C90.00, C90.02, C91.10, C91.11, C91.12, C91.40, C91.41, C91.Z0, C92.10, C92.11, C92.12, C92.50, C92.51, C92.90, C93.10, C93.12, C94.6, C95.00, C95.01, C95.02, C95.10, C95.90, D05.11, D45, D75.81, D47.1, R18.0
**Psychiatric conditions**	Depression, bipolar disorder, schizophrenia, schizoaffective disorder, phobias, obsessive-compulsive disorder, post-traumatic stress disorder, Alcohol dependent, opioid use, psychoactive substance use, stimulant use disorder	F10, F11, F15, F19, F20.0, F20.9, F25.0, F25.1, F20.9, F30.2, F30.3, F30.4, F30.9, F30.10, F30.12, F31.2, F31.4, F31.5, F31.9, F31.10, F31.12, F31.13, F31.30, F31.31, F31.32, F31.60, F31.62, F31.63, F31.64, F31.70, F31.71, F31.73, F31.74, F31.75 F31.76, F31.77, F31.81, F31.89, F32.1, F32.2, F33.0, F33.0, F33.1, F33.2, F33.3, F33.9, F33.40, F33.41, F33.42, F41.1, F42, F42.9, F43.10, F43.11, F43.12, F46.81, F60.1, F60.5

Supplement Table 2. Baseline characteristics of patients stratified by age: <70 vs. ≥70 years

Categories	Characteristics	Age <70 years, n=2221 (40%)	Age≥ 70 years, n=7035 (60%)	P value
**Demographics**	Age, years, mean ± SD	59.6 ± 9.6	83.5 ± 7.2	<0.0001
	Female, n= (%)	1050 (47)	3954 (56)	<0.0001
	Male, n= (%)	1171 (53)	3081 (44)	
	White, n= (%)	1985 (89)	6845 (97)	<0.0001
	Non-white, n= (%)	236 (11)	190 (3)	
**Social indicators**	Living with partner, n= (%)	1127 (51)	3131 (45)	<0.0001
	Not living with partner, n= (%)	1094 (49)	3904 (55)	
	Current smoker, n= (%)	504 (23)	462 (7)	<0.0001
	No current smoker, n= (%)	1717 (77)	6573 (93)	
	Rural	871 (39.2)	2846 (40.4)	0.3088
	Urban	1350 (60.8)	4189 (59.6)	
**Anthropometrics**	BMI, Kg/m^2, median (IQR)	34 (30 – 43)	30 (26 – 34)	<0.0001
**Pre-admission coronary angiogram PCI, devices, cardiac surgery**	Coronary angiogram	701 (31.5)	1761 (25.0)	<0.0001
	PCI	200 (9.0)	602 (8.6)	0.5165
	Pacemaker	82 (3.7)	651 (9.6)	<0.0001
	ICD	44 (2.0)	67 (1.0)	<0.0001
	LVAD	8 (0.4)	13 (0.2)	0.1311
	Valve repair or replacement	202 (9.1)	696 (9.9)	0.2850
	CABG	132 (5.9)	300 (4.2)	0.0011
**Vitals**	SBP, mmHg, mean ± SD	128 (22)	131 (19)	<0.0001
	DBP, mmHg, mean ± SD	78 (19)	76 (18)	<0.0001
	Heart rate, per min, mean ± SD	84 (26)	79 (25)	<0.0001
**Laboratory measures**	Sodium, mmol/L, mean ± SD	137 (5)	137 (5)	0.0280
	BUN, mg/dL, Median (IQR)	21 (15 – 35)	25 (18 – 37)	<0.0001
	Creatinine, mg/dL, Median (IQR)	1.0 (0.8 – 1.4)	1.1 (0.9 – 1.4)	0.0054
**Risk stratifications, GWTG**	1, n= (%)	700 (31.6)	555 (7.9)	<0.0001
	2, n= (%)	1418 (63.4)	5388 (76.7)	<0.0001
	3, n= (%)	86 (3.9)	857 (12.1)	<0.0001
	4 or more, n= (%)	17 (1.1)	235 (3.3)	<0.0001
**Admission service**	Internal Medicine, n= (%)	996 (44.8)	3975 (56.5)	<0.0001
	Critical care, n= (%)	159 (7.2)	314 (4.5)	<0.0001
	Specialty, n= (%)	1066 (47.9)	2746 (39.0)	<0.0001
**Length of stay**	Days, median (IQR)	4 (3 – 7)	4 (3 – 6)	<0.0001
**Medical treatment**	ACEI/ARBs/ARNI, n= (%)	1011 (45.5)	2527 (35.9)	<0.0001
	Beta-blocker, n= (%)	1175 (52.9)	2684 (38.1)	<0.0001
	MRA, n= (%)	694 (31.2)	1426 (20.3)	<0.0001
	SGLT2 inhibitors, n= (%)	227 (10.2)	296 (4.2)	<0.0001
	Diuretics, n= (%)	1065 (47.9)	2840 (40.4)	<0.0001
	Anticoagulation, n= (%)	793 (35.7)	2128 (30.2)	<0.0001
**Discharge destination**	Home, selfcare	1595 (71.8)	3630 (51.6)	<0.0001
	Home, home health	172 (7.7)	675 (9.6)	0.0042
	Rehabilitation	18 (0.8)	61 (0.9)	0.8950
	Nursing home	236 (10.6)	1956 (27.8)	<0.0001
	Other acute care	64 (2.9)	161 (2.3)	0.1145
	Hospice	44 (1.9)	317 (4.5)	<0.0001
	Other destinations	26 (1.2)	12 (0.2)	<0.0001
	Expired	66 (2.9)	223 (3.2)	0.6750

Abbreviations: ACEI/ARBs/ARNI, angiotensin-converting enzyme inhibitors/ angiotensin II receptor blockers/ angiotensin receptor-neprilysin inhibitors; BMI, body mass index; DBP, diastolic blood pressure; BUN, blood urea nitrogen; CABG, coronary artery bypass surgery; GWTG, get with the guideline; ICD, implantable cardiac defibrillator; IQR, interquartile range; LVAD, left ventricular assist device; LVEF, left ventricular ejection fraction; MRA, mineralocorticoid receptor antagonist; PCI, percutaneous coronary intervention; SD, standard deviation; SGLT2i, sodium-glucose cotransporter-2 inhibitors.

Supplement Table 3. Baseline characteristics of patients stratified by sex: female vs. male

Categories	Characteristics	Female, n=5004	Male, n=4252	P value
**Demographics**	Age, years, mean ± SD	79.1 ± 12.8	76.2 ± 12.8	<0.0001
	White, n= (%)	4766 (95.2)	4064 (95.6)	0.4437
	Non-white, n= (%)	238 (4.8)	188 (4.4)	
**Social indicators**	Living with partner, n= (%)	1611 (32.2)	2647 (62.3)	<0.0001
	Not living with partner, n= (%)	3393 (67.8)	1605 (37.7)	
	Current smoker, n= (%)	414 (8.3)	552 (12.9)	<0.0001
	No current smoker, n= (%)	4590 (91.7)	3700 (87.0)	
	Rural, n= (%)	2062 (41.2)	1655 (38.9)	0.0255
	Urban, n= (%)	2942 (58.8)	2597 (61.1)	
**Anthropometrics**	BMI, Kg/m^2, median (IQR)	30 (25 – 37)	30 (27 – 36)	0.0462
**Pre-admission coronary angiogram PCI, devices, cardiac surgery**	Coronary angiogram	1127 (22.5)	1335 (31.4)	<0.0001
	PCI	327 (6.5)	475 (11.1)	<0.0001
	Pacemaker	361 (7.1)	372 (8.7)	0.0068
	ICD	31 (0.6)	80 (1.9)	0.0387
	LVAD	6 (0.1)	15 (0.4)	0.0264
	Valve repair or replacement	410 (8.2)	488 (11.5)	<0.0001
	CABG	159 (3.2)	273 (6.4)	<0.0001
**Vitals**	SBP, mmHg, mean ± SD	131 ± 20	129 ±2 0	<0.0001
	DBP, mmHg, mean ± SD	76 ± 19	77 ± 18	0.5463
	Heart rate, per min, mean ± SD	81 ± 25	80 ± 25	0.0001
**Laboratory measures**	Sodium, mmol/L, mean ± SD	137 ± 5	137 ± 5	0.0020
	BUN, mg/dL, Median (IQR)	23 (16 – 35)	26 (19 – 39)	<0.0001
	Creatinine, mg/dL, Median (IQR)	0.9 (0.8 – 1.29)	1.18 (0.9 – 1.54)	<0.0001
**Risk stratifications, GWTG**	1, n= (%)	679 (13.6)	576 (13.5)	0.9748
	2, n= (%)	3731 (75.6)	3075 (72.3)	0.0149
	3, n= (%)	480 (9.6)	463 (10.9)	0.0419
	4 or more, n= (%)	114 (2.3)	138 (3.2)	0.0048
**Admission service**	Internal Medicine, n= (%)	2871 (57.4)	2100 (49.4)	<0.0001
	Critical care, n= (%)	233 (4.7)	240 (5.6)	0.0331
	Specialty, n= (%)	1900 (37.9)	1912 (44.9)	<0.0001
**Length of stay**	Days, median (IQR)	4 (3 – 6)	4 (3 – 6)	0.7091
**Medical treatment**	ACEI/ARBs/ARNI, n= (%)	1926 (38.5)	162 (37.9)	0.5769
	Beta-blocker, n= (%)	2099 (38.5)	1760 (41.4)	0.5970
	MRA, n= (%)	1148 (22.9)	972 (22.9)	0.9407
	SGLT2i, n= (%)	243 (4.9)	280 (6.6)	0.0004
	Diuretics, n= (%)	2120 (42.4)	1785 (41.9)	0.7196
	Anticoagulation, n= (%)	1527 (30.5)	1394 (32.8)	0.0196
**Discharge destination**	Home, selfcare	2617 (52.3)	2608 (61.3)	<0.0001
	Home, home health	473 (9.5)	374 (8.8)	0.2781
	Rehabilitation	31 (0.6)	48 (1.1)	0.0089
	Nursing home	1383 (27.6)	809 (19.0)	<0.0001
	Other acute care	119 (2.3)	106 (2.5)	0.7352
	Hospice	200 (3.9)	161 (3.8)	0.6279
	Other destinations	14 (0.3)	24 (0.6)	0.0348
	Expired	167 (3.4)	122 (2.9)	0.0255

Abbreviations: ACEI/ARBs/ARNI, angiotensin-converting enzyme inhibitors/ angiotensin II receptor blockers/ angiotensin receptor-neprilysin inhibitors; BMI, body mass index; DBP, diastolic blood pressure; BUN, blood urea nitrogen; CABG, coronary artery bypass surgery; GWTG, get with the guideline; ICD, implantable cardiac defibrillator; IQR, interquartile range; LVAD, left ventricular assist device; LVEF, left ventricular ejection fraction; MRA, mineralocorticoid receptor antagonist; PCI, percutaneous coronary intervention; SD, standard deviation; SGLT2i, sodium-glucose cotransporter-2 inhibitors.

Table 4. Baseline characteristics of patients stratified by residential status: rural vs. urban

Categories	Characteristics	Rural, n=3717 (40%)	Urban, n=5539 (60%)	P value
Demographics	Age, years, mean ± SD	77.9 ± 12.5	77.6 ± 13.1	0.2335
	Age, <70, n= (%)	871 (23.4)	1350 (24.3)	0.3380
	Age, ≥70, n= (%)	2846 (76.6)	4189 (75.6)	
	Female, n= (%)	2062 (55.5)	2942 (53.1)	0.0362
	Male, n= (%)	1655 (44.5)	2597 (46.9)	
	White, n= (%)	3610 (97.1)	5220 (94.2)	<0.0001
	Non-white, n= (%)	107 (28.9)	319 (5.8)	
Social indicators	Married, n= (%)	1676 (45.1)	2582 (46.6)	0.1857
	Nonmarried, n= (%)	2041 (54.9)	2957 (53.4)	
	Current smoker, n= (%)	445 (12.0)	521 (9.4)	0.0001
	Non- or ex-smoker, n= (%)	3272 (88.0)	5018 (90.6)	
Anthropometrics	BMI, Kg/m^2, median (IQR)	31.0 (25.9-38.2)	29.9 (25.4-35.9)	<0.0001
Pre-admission coronary angiogram PCI, devices, cardiac surgery	Coronary angiogram	947 (25.5)	1515 (27.4)	0.0235
	PCI	324 (8.7)	478 (8.6)	0.9702
	Pacemaker	284 (7.6)	449 (8.2)	0.5107
	ICD	41 (1.1)	70 (1.3)	0.5057
	LVAD	7 (0.2)	14 (0.3)	0.2292
	Valve repair or replacement	333 (9.0)	565 (10.2)	0.0305
	CABG	175 (4.7)	257 (4.6)	0.8421
Vitals	SBP, mmHg, mean ± SD	136 ± 24	137 ± 25	0.0094
	DBP, mmHg, mean ± SD	76 ± 19	77 ± 18	0.0375
	Heart rate, per min, mean ± SD	80 ± 24	81 ± 26	0.0829
Laboratory measures	Sodium, mmol/L, mean ± SD	137.1 ± 5.2	136.9 ± 5.1	0.2804
	BUN, mg/dL, Median (IQR)	24.0 (17.0-37.0)	24.2 (17.0-36.0)	0.4972
	Creatinine, mg/dL, Median (IQR)	1.01 (0.83-1.34)	1.10 (0.86-1.45)	0.0734
Risk stratifications, GWTG	1, n= (%)	473 (12.7)	782 (14.1)	0.0589
	2, n= (%)	2762 (74.3)	4044 (73.0)	0.1708
	3, n= (%)	365 (9.8)	578 (10.4)	0.3442
	4 or more, n= (%)	117 (3.1)	135 (2.4)	0.0433
Admission service	Internal Medicine, n= (%)	2288 (61.6)	2683 (48.4)	<0.0001
	Critical care, n= (%)	260 (7.0)	213 (3.8)	<0.0001
	Specialty, n= (%)	1169 (31.4)	2643 (47.7)	<0.0001
Length of stay	Days, median (IQR)	4 (3-6)	4 (3-7)	0.0176
Medical treatment	ACEI/ARBs/ARNI, n= (%)	1421 (38.2)	2117 (38.2)	0.9924
	Beta-blocker, n= (%)	1522 (40.9)	2337 (42.2)	0.2370
	MRA, n= (%)	818 (22.0)	1302(23.5)	0.0959
	SGLT2 inhibitors, n= (%)	168 (4.5)	355 (6.4)	0.0001
	Diuretics, n= (%)	1440 (38.7)	2465 (44.5)	<0.0001
	Anticoagulation, n= (%)	1101 (29.6)	1820 (32.9)	0.0010
Discharge destination	Home, selfcare	2105 (56.6)	3120 (56.3)	0.7811
	Home, home health	204 (5.4)	643 (11.6)	<0.0001
	Rehabilitation	19 (0.5)	60 (1.1)	0.0037
	Nursing home	992 (26.7)	1200 (21.7)	<0.0001
	Other acute care	120 (3.2)	105 (1.9)	<0.0001
	Hospice	122 (3.2)	239 (4.3)	0.0118
	Other destinations	15 (0.4)	23 (0.4)	0.9313
	Expired	140 (3.8)	149 (2.6)	0.0041

Abbreviations: ACEI/ARBs/ARNI, angiotensin-converting enzyme inhibitors/ angiotensin II receptor blockers/ angiotensin receptor-neprilysin inhibitors; BMI, body mass index; DBP, diastolic blood pressure; BUN, blood urea nitrogen; CABG, coronary artery bypass surgery; GWTG, get with the guideline; ICD, implantable cardiac defibrillator; IQR, interquartile range; LVAD, left ventricular assist device; LVEF, left ventricular ejection fraction; MRA, mineralocorticoid receptor antagonist; PCI, percutaneous coronary intervention; SD, standard deviation; SGLT2i, sodium-glucose cotransporter-2 inhibitors.

Supplement Figure 2. Heat Map Showing Visual Representation of Pairwise Correlations Between Comorbidities.

	HTN	CAD	Afib	VHD	Stroke	HLP	DM	Obesity	CLD	GI	CKD	Rheum	Anemia	Cancer	Psych	Neuro	VIF
HTN	1	0.265	0.241	0.103	0.186	0.63	0.266	0.267	0.248	0.148	0.205	0.31	0.095	0.19	0.254	0.149	1.86
CAD	0.265	1	0.226	0.232	0.13	0.287	0.197	0.089	0.147	0.119	0.151	0.187	0.065	0.133	0.113	0.097	1.22
Afib	0.241	0.226	1	0.207	0.108	0.22	0.078	0.086	0.099	0.118	0.099	0.185	0.045	0.127	0.059	0.076	1.15
VHD	0.103	0.232	0.207	1	0.068	0.122	0.02	0.043	0.044	0.064	0.06	0.082	0.024	0.074	0.029	0.057	1.09
Stroke	0.186	0.13	0.108	0.068	1	0.189	0.094	0.087	0.082	0.079	0.055	0.082	0.02	0.085	0.116	0.177	1.08
HLP	0.63	0.287	0.22	0.122	0.189	1	0.288	0.253	0.218	0.129	0.206	0.275	0.08	0.154	0.215	0.123	1.79
DM	0.266	0.197	0.078	0.02	0.094	0.288	1	0.219	0.115	0.102	0.178	0.181	0.07	0.082	0.175	0.054	1.18
Obesity	0.267	0.089	0.086	0.043	0.087	0.253	0.219	1	0.176	0.056	0.079	0.174	0.024	0.082	0.18	0.038	1.14
CLD	0.248	0.147	0.099	0.044	0.082	0.218	0.115	0.176	1	0.117	0.084	0.151	0.032	0.126	0.199	0.079	1.12
GI	0.148	0.119	0.118	0.064	0.079	0.129	0.102	0.056	0.117	1	0.088	0.081	0.121	0.149	0.137	0.048	1.08
CKD	0.205	0.151	0.099	0.06	0.055	0.206	0.178	0.079	0.084	0.088	1	0.112	0.114	0.086	0.053	0.055	1.09
Rheum	0.31	0.187	0.185	0.082	0.082	0.275	0.181	0.174	0.151	0.081	0.112	1	0.084	0.145	0.166	0.079	1.17
Anemia	0.095	0.065	0.045	0.024	0.02	0.08	0.07	0.024	0.032	0.121	0.114	0.084	1	0.066	0.086	0.073	1.04
Cancer	0.19	0.133	0.127	0.074	0.085	0.154	0.082	0.082	0.126	0.149	0.086	0.145	0.066	1	0.098	0.04	1.08
Psych	0.254	0.113	0.059	0.029	0.116	0.215	0.175	0.18	0.199	0.137	0.053	0.166	0.086	0.098	1	0.178	1.16
Neuro	0.149	0.097	0.076	0.057	0.177	0.123	0.054	0.038	0.079	0.048	0.055	0.079	0.073	0.04	0.178	1	1.08

Low
Mid
High

Each variable is represented by a row and a column. The colored cells indicating the strength of correlation, reddish hues represent stronger correlations and bluish hues represent weaker correlations. The Variance Inflation Factor (VIF) is displayed in the right column, with each comorbidity variable represented by a gray cell. VIF values ranged from 1.02 to 1.30, well below the threshold of 10, indicating no significant multicollinearity and confirming that the predictors were appropriate for inclusion in the multivariable model.

Abbreviations: Afib, atrial fibrillation; CAD, coronary artery disease; CLD, chronic lung disease; CKD, chronic kidney disease ≥ stage 3; DM, diabetes mellitus; GI, gastrointestinal/hepatic conditions; HLP, hyperlipidemia; HTN, hypertension; Neuro, non-stroke neurological condition; Psych, psychiatric condition; Rheum, rheumatological condition; VHD, valvular heart disease.

Supplement Figure 3A. Adjusted Hazard Ratios and 95% Confidence Intervals for Mortality by Comorbidity Duration in Patients Aged < 70 Years, Displayed as a Forest Plot.

Comorbidities		HR (95% CI)	P value
Hypertension	No hypertension	Reference	
	Model I, <0.5y	1.28 (1.17 – 1.40)	<0.0001
	Model II, <0.5 - <3y	1.59 (1.34 – 1.83)	<0.0001
	Model III, 3 - <6y	1.63 (1.41 – 1.90)	<0.0001
	Model IV, >6y	1.63 (1.31 – 2.03)	<0.0001
CAD	No CAD	Reference	
	Model I, <0.5y	1.34 (1.21 – 1.49)	<0.0001
	Model II, <0.5 - <3y	1.70 (1.43 – 2.02)	<0.0001
	Model III, 3 - <6y	1.73 (1.49 – 2.00)	<0.0001
	Model IV, >6y	1.66 (1.34 – 2.06)	<0.0001
Atrial fibrillation	No atrial fibrillation	Reference	
	Model I, <0.5y	1.23 (1.10 – 1.36)	0.0001
	Model II, <0.5 - <3y	1.47 (1.23 – 1.75)	<0.0001
	Model III, 3 - <6y	1.60 (1.35 – 1.91)	<0.0001
	Model IV, >6y	1.71 (1.28 – 2.30)	0.0003
VHD	No VHD	Reference	
	Model I, <0.5y	1.22 (1.04 – 1.42)	0.0120
	Model II, <0.5 - <3y	1.39 (1.08 – 1.79)	0.0105
	Model III, 3 - <6y	1.25 (0.99 – 1.56)	0.0540
	Model IV, >6y	1.05 (0.73 – 1.52)	0.7788
Stroke	No stroke	Reference	
	Model I, <0.5y	1.33 (1.14 – 1.56)	0.0003
	Model II, <0.5 - <3y	1.62 (1.26 – 2.09)	0.0002
	Model III, 3 - <6y	1.37 (1.04 – 1.81)	0.0276
	Model IV, >6y	1.06 (0.62 – 1.80)	0.8369
Hyperlipidemia	No hyperlipidemia	Reference	
	Model I, <0.5y	1.34 (1.22 – 1.47)	<0.0001
	Model II, <0.5 - <3y	1.69 (1.45 – 1.97)	<0.0001
	Model III, 3 - <6y	1.67 (1.43 – 1.96)	<0.0001
	Model IV, >6y	1.56 (1.21 – 2.00)	0.0006
DM	No DM	Reference	
	Model I, <0.5y	1.04 (0.94 – 1.15)	0.4797
	Model II, <0.5 - <3y	1.10 (0.93 – 1.32)	0.2709
	Model III, 3 - <6y	1.29 (1.12 – 1.49)	0.0003
	Model IV, >6y	1.55 (1.33 – 1.81)	<0.0001
Obesity	No obesity	Reference	
	Model I, <0.5y	0.98 (0.87 – 1.10)	0.7006
	Model II, <0.5 - <3y	0.97 (0.81 – 1.17)	0.7720
	Model III, 3 - <6y	1.04 (0.78 – 1.40)	0.7849
	Model IV, >6y	1.14 (0.92 – 2.09)	0.6817
CKD	No CAD	Reference	
	Model I, <0.5y	1.37 (1.21 – 1.54)	<0.0001
	Model II, <0.5 - <3y	1.80 (1.48 – 2.19)	<0.0001
	Model III, 3 - <6y	2.04 (1.64 – 2.54)	<0.0001
	Model IV, >6y	2.23 (1.48 – 3.35)	0.0001
Anemia	No anemia	Reference	
	Model I, <0.5y	1.38 (1.17 – 1.62)	0.0001
	Model II, <0.5 - <3y	1.79 (1.37 – 2.35)	<0.0001
	Model III, 3 - <6y	1.86 (1.48 – 2.36)	<0.0001
	Model IV, >6y	1.83 (1.25 – 2.70)	0.0020
CLC	No CLC	Reference	
	Model I, <0.5y	1.37 (1.21 – 1.54)	<0.0001
	Model II, <0.5 - <3y	1.80 (1.48 – 2.19)	<0.0001
	Model III, 3 - <6y	2.04 (1.64 – 2.54)	<0.0001
	Model IV, >6y	2.23 (1.48 – 3.35)	0.0001
GI/hepatic	No GI/hepatic	Reference	
	Model I, <0.5y	1.34 (1.19 – 1.52)	<0.0001
	Model II, <0.5 - <3y	1.69 (1.38 – 2.06)	<0.0001
	Model III, 3 - <6y	1.60 (1.34 – 1.93)	<0.0001
	Model IV, >6y	1.42 (1.05 – 1.93)	0.0217
Cancer	No cancer	Reference	
	Model I, <0.5y	1.44 (1.29 – 1.62)	<0.0001
	Model II, <0.5 - <3y	1.90 (1.57 – 2.29)	<0.0001
	Model III, 3 - <6y	1.73 (1.46 – 2.05)	<0.0001
	Model IV, >6y	1.44 (1.09 – 1.90)	0.0109
Rheum	No rheum	Reference	
	Model I, <0.5y	1.15 (1.04 – 1.28)	0.0091
	Model II, <0.5 - <3y	1.32 (1.10 – 1.57)	0.0024
	Model III, 3 - <6y	1.45 (1.24 – 1.69)	<0.0001
	Model IV, >6y	1.57 (1.23 – 2.00)	0.0003
Neurological	No neurological	Reference	
	Model I, <0.5y	1.33 (1.14 – 1.55)	0.0004
	Model II, <0.5 - <3y	1.61 (1.25 – 2.08)	0.0002
	Model III, 3 - <6y	1.38 (1.05 – 1.79)	0.0193
	Model IV, >6y	1.07 (0.65 – 1.78)	0.7841
Psychiatric	No psychiatric	Reference	
	Model I, <0.5y	1.11 (1.01 – 1.23)	0.0371
	Model II, <0.5 - <3y	1.23 (1.04 – 1.46)	0.0174
	Model III, 3 - <6y	1.32 (1.15 – 1.52)	0.0001
	Model IV, >6y	1.41 (1.19 – 1.67)	<0.0001

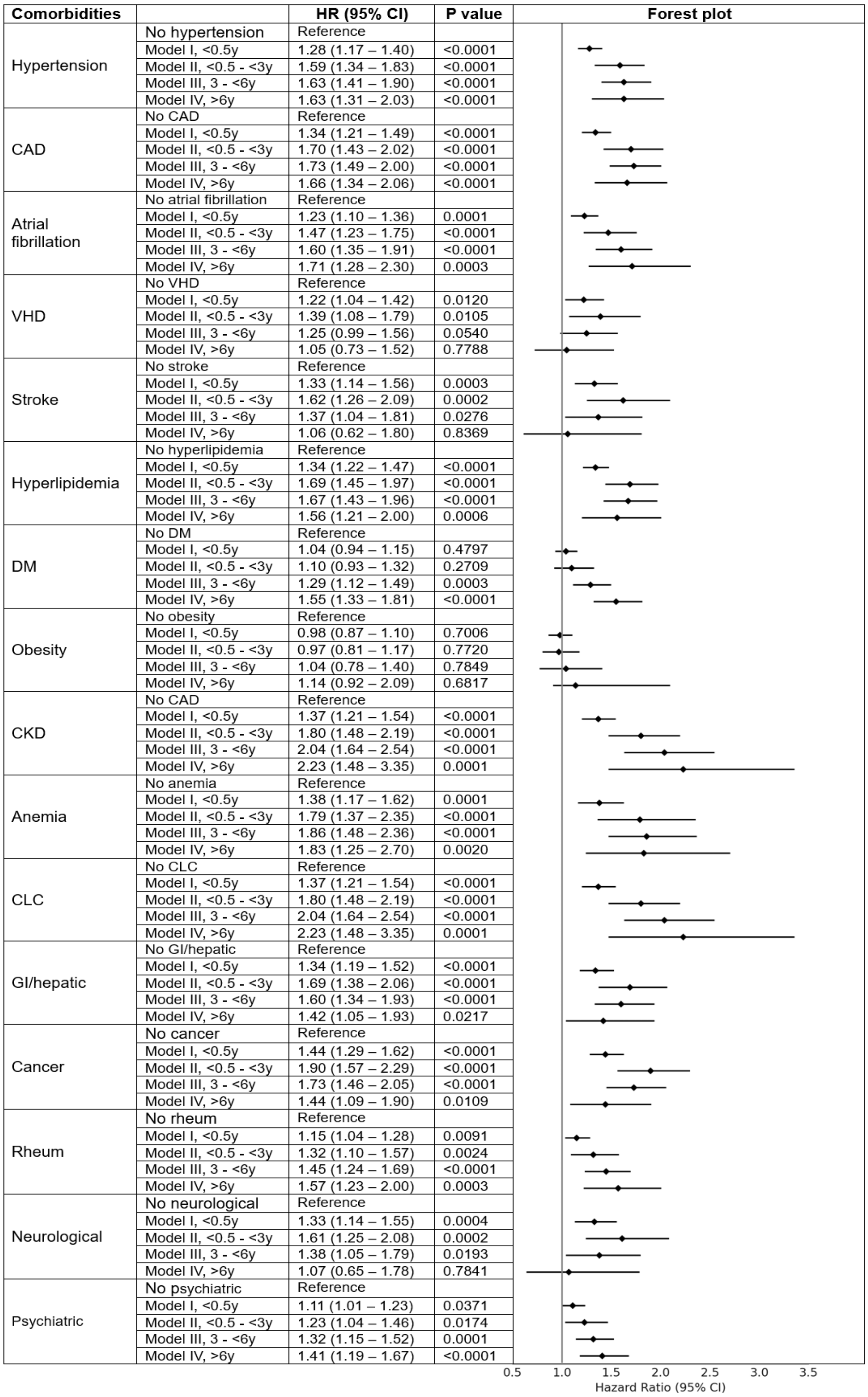

Model I: Multivariable Cox regression model estimating mortality based on preadmission comorbidity duration of < 0.5 years.
Model II: Multivariable Cox regression model estimating mortality based on preadmission comorbidity duration of 0.5 to < 3 years.
Model III: Multivariable Cox regression model estimating mortality based on preadmission comorbidity duration of 3 to < 6 years.
Model IV: Multivariable Cox regression model estimating mortality based on preadmission comorbidity duration of ≥ 6 years.

Most comorbidities demonstrated an increase in effect size (HR) as the duration extended from < 0.5 years to 0.5 to < 3 years, followed by either a slower increase, a plateau, or even a decline once the comorbidity duration reached 3 years or more.

Abbreviations: CAD, coronary artery disease; CLC, chronic lung condition; CKD, chronic kidney disease ≥ stage 3; DM, diabetes mellitus; GI/hepatic, gastrointestinal/hepatic conditions; Neurological, non-stroke neurological condition; Psychiatric, psychiatric condition; Rheum, rheumatological condition; VHD, valvular heart disease.

Supplement Figure 3B. Adjusted Hazard Ratios and 95% Confidence Intervals for Mortality by Comorbidity Duration in Patients Aged ≥70 Years, Displayed as a Forest Plot.

Comorbidities		HR (95% CI)	P value	Forest plot
Hypertension	No hypertension	Reference		
	Model I, <0.5y	1.31 (1.25 – 1.37)	<0.0001	
	Model II, <0.5 - <3y	1.62 (1.50 – 1.74)	<0.0001	
	Model III, 3 - <6y	1.59 (1.48 – 1.71)	<0.0001	
	Model IV, >6y	1.48 (1.35 – 1.63)	<0.0001	
CAD	No CAD	Reference		
	Model I, <0.5y	1.14 (1.09 – 1.20)	<0.0001	
	Model II, <0.5 - <3y	1.28 (1.19 – 1.38)	<0.0001	
	Model III, 3 - <6y	1.33 (1.24 – 1.41)	<0.0001	
	Model IV, >6y	1.34 (1.25 – 1.45)	<0.0001	
Atrial fibrillation	No atrial fibrillation	Reference		
	Model I, <0.5y	1.15 (1.10 – 1.20)	<0.0001	
	Model II, <0.5 - <3y	1.29 (1.20 – 1.39)	<0.0001	
	Model III, 3 - <6y	1.36 (1.28 – 1.45)	<0.0001	
	Model IV, >6y	1.41 (1.29 – 1.54)	<0.0001	
VHD	No VHD	Reference		
	Model I, <0.5y	1.08 (1.02 – 1.15)	0.0082	
	Model II, <0.5 - <3y	1.17 (1.06 – 1.29)	0.0017	
	Model III, 3 - <6y	1.25 (1.15 – 1.37)	<0.0001	
	Model IV, >6y	1.34 (1.17 – 1.54)	<0.0001	
Stroke	No stroke	Reference		
	Model I, <0.5y	1.18 (1.11 – 1.25)	<0.0001	
	Model II, <0.5 - <3y	1.34 (1.21 – 1.48)	<0.0001	
	Model III, 3 - <6y	1.32 (1.20 – 1.45)	<0.0001	
	Model IV, >6y	1.24 (1.05 – 1.47)	0.0136	
Hyperlipidemia	No hyperlipidemia	Reference		
	Model I, <0.5y	1.18 (1.13 – 1.23)	<0.0001	
	Model II, <0.5 - <3y	1.35 (1.26 – 1.45)	<0.0001	
	Model III, 3 - <6y	1.36 (1.27 – 1.46)	<0.0001	
	Model IV, >6y	1.34 (1.21 – 1.47)	<0.0001	
DM	No hyperlipidemia	Reference		
	Model I, <0.5y	1.02 (0.97 – 1.08)	0.3623	
	Model II, <0.5 - <3y	1.05 (0.96 – 1.15)	0.2564	
	Model III, 3 - <6y	1.09 (1.02 – 1.17)	0.0092	
	Model IV, >6y	1.13 (1.06 – 1.21)	0.0003	
Obesity	No obesity	Reference		
	Model I, <0.5y	0.89 (0.84 – 0.95)	0.0004	
	Model II, <0.5 - <3y	0.84 (0.76 – 0.93)	0.0005	
	Model III, 3 - <6y	0.96 (0.84 – 1.10)	0.5874	
	Model IV, >6y	1.16 (0.88 – 1.52)	0.2887	
CKD	No CAD	Reference		
	Model I, <0.5y	1.31 (1.25 – 1.37)	<0.0001	
	Model II, <0.5 - <3y	1.63 (1.51 – 1.76)	<0.0001	
	Model III, 3 - <6y	1.64 (1.50 – 1.79)	<0.0001	
	Model IV, >6y	1.56 (1.32 – 185)	<0.0001	
Anemia	No anemia	Reference		
	Model I, <0.5y	1.24 (1.16 – 1.32)	<0.0001	
	Model II, <0.5 - <3y	1.47 (1.32 – 1.64)	<0.0001	
	Model III, 3 - <6y	1.47 (1.34 – 1.62)	<0.0001	
	Model IV, >6y	1.42 (1.21 – 1.67)	<0.0001	
CLC	No CLC	Reference		
	Model I, <0.5y	1.18 (1.13 – 1.24)	<0.0001	
	Model II, <0.5 - <3y	1.33 (1.24 – 1.44)	<0.0001	
	Model III, 3 - <6y	1.23 (1.13 – 1.34)	<0.0001	
	Model IV, >6y	1.09 (0.92 – 1.28)	0.3229	
GI/hepatic	No GI/hepatic	Reference		
	Model I, <0.5y	1.21 (1.14 – 1.24)	<0.0001	
	Model II, <0.5 - <3y	1.38 (1.26 – 1.52)	<0.0001	
	Model III, 3 - <6y	1.27 (1.17 – 1.38)	<0.0001	
	Model IV, >6y	1.10 (0.97 – 1.25)	0.1441	
Cancer	No cancer	Reference		
	Model I, <0.5y	1.21 (1.15 – 127)	<0.0001	
	Model II, <0.5 - <3y	1.40 (1.29 – 1.52)	<0.0001	
	Model III, 3 - <6y	1.35 (1.26 – 1.44)	<0.0001	
	Model IV, >6y	1.24 (1.13 – 1.36)	<0.0001	
Rheum	No rheum	Reference		
	Model I, <0.5y	1.16 (1.11 – 1.21)	<0.0001	
	Model II, <0.5 - <3y	1.29 (1.20 – 1.39)	<0.0001	
	Model III, 3 - <6y	1.25 (1.17 – 1.33)	<0.0001	
	Model IV, >6y	1.17 (1.07 – 1.27)	0.0004	
Neurological	No neurological			
	Model I, <0.5y	1.34 (1.27 – 1.42)	<0.0001	
	Model II, <0.5 - <3y	1.68 (1.53 – 1.84)	<0.0001	
	Model III, 3 - <6y	1.58 (1.43 – 1.75)	<0.0001	
	Model IV, >6y	1.39 (1.14 – 1.69)	0.0011	
Psychiatric	No psychiatric	Reference		
	Model I, <0.5y	1313 (1.08 – 1.20)	<0.0001	
	Model II, <0.5 - <3y	1.25 (1.15 – 1.36)	<0.0001	
	Model III, 3 - <6y	1.13 (1.13 – 1.29)	<0.0001	
	Model IV, >6y	1.12 (1.03 – 1.23)	0.0088	

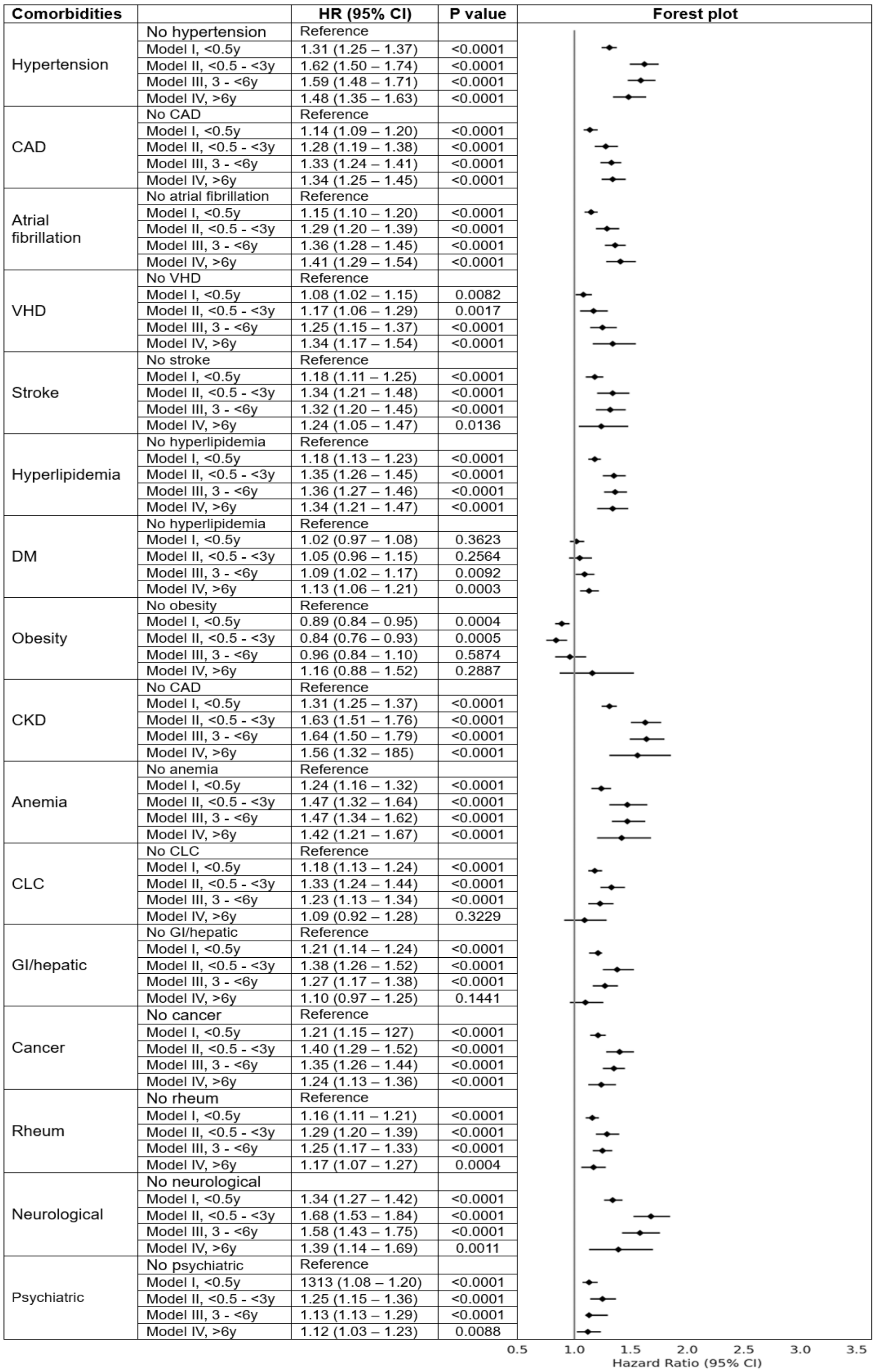

Model I: Multivariable Cox regression model estimating mortality based on preadmission comorbidity duration of < 0.5 years.
Model II: Multivariable Cox regression model estimating mortality based on preadmission comorbidity duration of 0.5 to < 3 years.
Model III: Multivariable Cox regression model estimating mortality based on preadmission comorbidity duration of 3 to < 6 years.
Model IV: Multivariable Cox regression model estimating mortality based on preadmission comorbidity duration of ≥ 6 years.

Most comorbidities demonstrated an increase in effect size (HR) as the duration extended from < 0.5 years to 0.5 to < 3 years, followed by either a slower increase, a plateau, or even a decline once the comorbidity duration reached 3 years or more.

Abbreviations: CAD, coronary artery disease; CLC, chronic lung condition; CKD, chronic kidney disease ≥ stage 3; DM, diabetes mellitus; GI/hepatic, gastrointestinal/hepatic conditions; Neurological, non-stroke neurological condition; Psychiatric, psychiatric condition; Rheum, rheumatological condition; VHD, valvular heart disease.

Supplement Figure 4A. Adjusted Hazard Ratios and 95% Confidence Intervals for Mortality by Comorbidity Duration in Female Patients, Displayed as a Forest Plot.

Comorbidities		HR (95% CI)	P value	Forest plot
Hypertension	No hypertension	Reference		
	Model I, <0.5y	1.38 (1.31 – 1.45)	<0.0001	
	Model II, <0.5 - <3y	1.78 (1.63 – 1.95)	<0.0001	
	Model III, 3 - <6y	1.74 (1.59 – 1.90)	<0.0001	
	Model IV, >6y	1.58 (1.41 – 178)	<0.0001	
CAD	No CAD	Reference		
	Model I, <0.5y	1.22 (1.15 – 1.29)	<0.0001	
	Model II, <0.5 - <3y	1.42 (1.29 – 156)	<0.0001	
	Model III, 3 - <6y	1.42 (1.32 – 1.54)	<0.0001	
	Model IV, >6y	1.37 (1.24 – 1.52)	<0.0001	
Atrial fibrillation	No atrial fibrillation	Reference		
	Model I, <0.5y	1.22 (1.16 – 1.29)	<0.0001	
	Model II, <0.5 - <3y	1.44 (132 – 1.58)	<0.0001	
	Model III, 3 - <6y	1.47 (1.35 – 1.59)	<0.0001	
	Model IV, >6y	1.44 (1.28 – 1.63)	<0.0001	
VHD	No VHD	Reference		
	Model I, <0.5y	1.14 (1.05 – 1.23)	0.0012	
	Model II, <0.5 - <3y	1.26 (1.11 – 1.44)	0.0005	
	Model III, 3 - <6y	1.26 (1.12 – 1.41)	0.0001	
	Model IV, >6y	1.22 (1.02 – 1.47)	0.0334	
Stroke	No stroke	Reference		
	Model I, <0.5y	1.19 (1.10 – 1.29)	<0.0001	
	Model II, <0.5 - <3y	1.37 (1.21 – 1.55)	<0.0001	
	Model III, 3 - <6y	1.35 (1.19 – 1.52)	<0.0001	
	Model IV, >6y	1.27 (1.02 – 1.59)	0.0361	
Hyperlipidemia	No hyperlipidemia	Reference		
	Model I, <0.5y	1.25 (1.19 – 1.32)	<0.0001	
	Model II, <0.5 - <3y	1.50 (1.37 (1.63)	<0.0001	
	Model III, 3 - <6y	1.48 (1.36 – 1.61)	<0.0001	
	Model IV, >6y	1.40 (1.24 – 1.59)	<0.0001	
DM	No hyperlipidemia	Reference		
	Model I, <0.5y	0.99 (0.92 – 1.05)	0.6517	
	Model II, <0.5 - <3y	0.99 (0.88 – 1.10)	0.8315	
	Model III, 3 - <6y	1.06 (0.98 – 1.15)	0.1275	
	Model IV, >6y	1.17 (1.07 – 1.27)	0.0004	
Obesity	No obesity	Reference		
	Model I, <0.5y	0.86 (0.80 – 0.93)	0.0001	
	Model II, <0.5 - <3y	0.78 (0.69 – 0.88)	<0.0001	
	Model III, 3 - <6y	0.88 (0.73 – 1.06)	0.1783	
	Model IV, >6y	1.04 (0.71 – 1.54)	0.8301	
CKD	No CAD	Reference		
	Model I, <0.5y	1.40 (1.32 – 1.50)	<0.0001	
	Model II, <0.5 - <3y	1.82 (1.64 – 2.02)	<0.0001	
	Model III, 3 - <6y	1.70 (1.51 – 1.93)	<0.0001	
	Model IV, >6y	1.48 (1.16 – 1.88)	0.0014	
Anemia	No anemia	Reference		
	Model I, <0.5y	1.31 (1.21 – 1.41)	<0.0001	
	Model II, <0.5 - <3y	1.62 (1.43 – 1.83)	<0.0001	
	Model III, 3 - <6y	1.60 (1.44 – 1.79)	<0.0001	
	Model IV, >6y	1.50 (1.44 – 1.79)	<0.0001	
CLC	No CLC	Reference		
	Model I, <0.5y	1.16 (1.10 – 1.23)	<0.0001	
	Model II, <0.5 - <3y	1.31 (1.19 – 1.43)	<0.0001	
	Model III, 3 - <6y	1.28 (1.15 – 1.42)	<0.0001	
	Model IV, >6y	1.24 (0.99 – 1.48)	0.0600	
GI/hepatic	No GI/hepatic	Reference		
	Model I, <0.5y	1.18 (1.10 – 1.27)	<0.0001	
	Model II, <0.5 - <3y	1.33 (1.18 – 1.50)	<0.0001	
	Model III, 3 - <6y	1.27 (1.14 – 1.41)	<0.0001	
	Model IV, >6y	1.16 (0.98 – 1.36)	0.0805	
Cancer	No cancer	Reference		
	Model I, <0.5y	1.19 (1.11 – 1.27)	<0.0001	
	Model II, <0.5 - <3y	1.36 (1.22 – 1.51)	<0.0001	
	Model III, 3 - <6y	1.31 (1.20 – 1.43)	<0.0001	
	Model IV, >6y	1.21 (1.07 – 1.38)	0.0027	
Rheum	No rheum	Reference		
	Model I, <0.5y	1.17 (1.11 – 1.24)	<0.0001	
	Model II, <0.5 - <3y	1.32 (1.20 – 1.45)	<0.0001	
	Model III, 3 - <6y	1.29 (1.20 – 1.40)	<0.0001	
	Model IV, >6y	1.22 (1.10 – 1.36)	0.0002	
Neurological	No neurological	Reference		
	Model I, <0.5y	1.46 (1.37 – 1.57)	<0.0001	
	Model II, <0.5 - <3y	1.93 (1.72 – 2.15)	<0.0001	
	Model III, 3 - <6y	1.67 (1.47 – 1.89)	<0.0001	
	Model IV, >6y	1.30 (1.02 – 1.65)	0.0350	
Psychiatric	No psychiatric	Reference		
	Model I, <0.5y	1.06 (0.99 – 1.12)	0.0676	
	Model II, <0.5 - <3y	1.10 (0.99 – 1.22)	0.0532	
	Model III, 3 - <6y	1.10 (1.01 – 1.19)	0.0231	
	Model IV, >6y	1.08 (0.97 – 1.19)	0.1454	

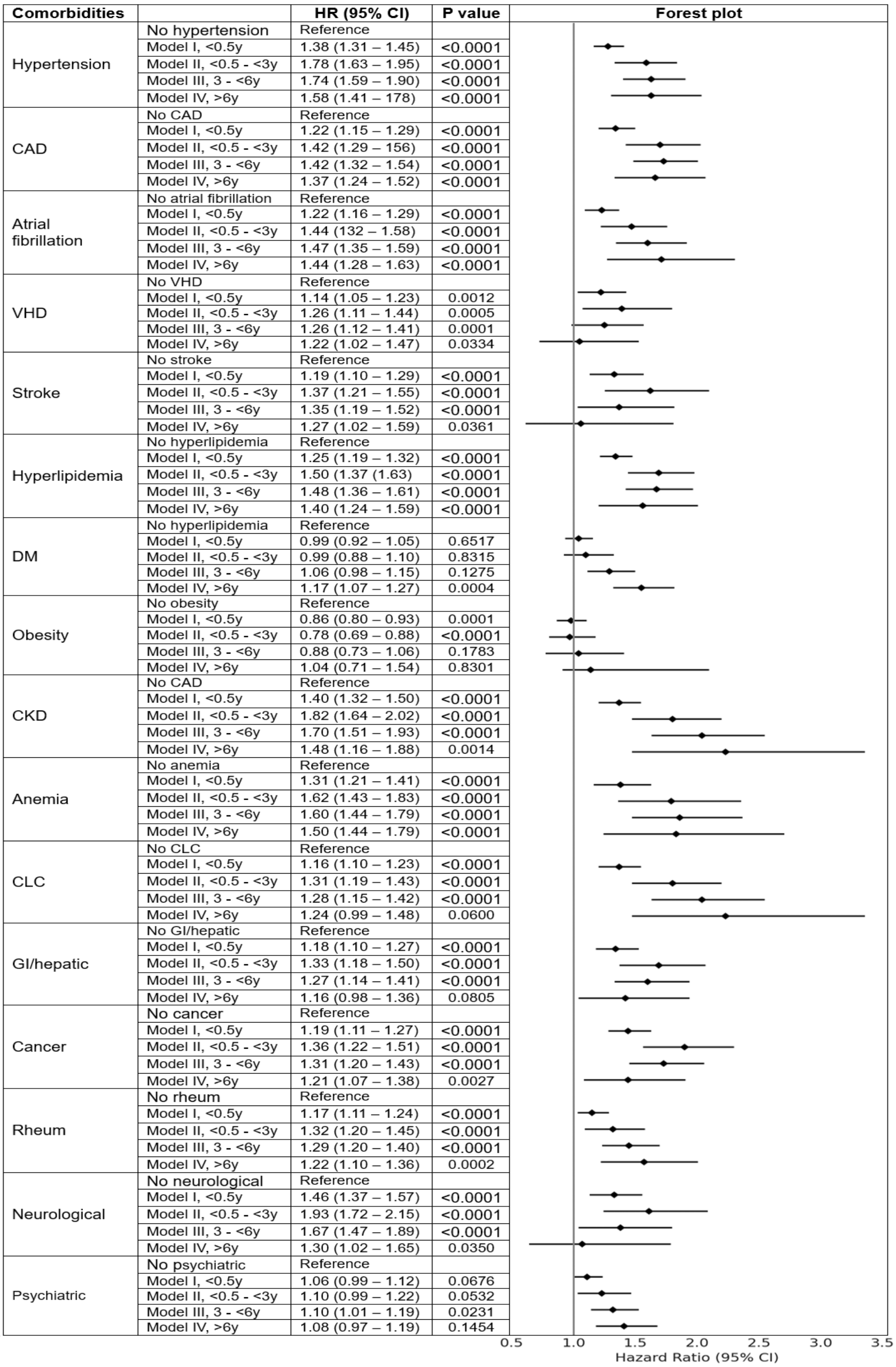

Model I: Multivariable Cox regression model estimating mortality based on preadmission comorbidity duration of < 0.5 years.
Model II: Multivariable Cox regression model estimating mortality based on preadmission comorbidity duration of 0.5 to < 3 years.
Model III: Multivariable Cox regression model estimating mortality based on preadmission comorbidity duration of 3 to < 6 years.
Model IV: Multivariable Cox regression model estimating mortality based on preadmission comorbidity duration of ≥ 6 years.

Most comorbidities demonstrated an increase in effect size (HR) as the duration extended from < 0.5 years to 0.5 to < 3 years, followed by either a slower increase, a plateau, or even a decline once the comorbidity duration reached 3 years or more.

Abbreviations: CAD, coronary artery disease; CLC, chronic lung condition; CKD, chronic kidney disease ≥ stage 3; DM, diabetes mellitus; GI/hepatic, gastrointestinal/hepatic conditions; Neurological, non-stroke neurological condition; Psychiatric, psychiatric condition; Rheum, rheumatological condition; VHD, valvular heart disease.

Supplement Figure 4B. Adjusted Hazard Ratios and 95% Confidence Intervals for Mortality by Comorbidity Duration in Male Patients, Displayed as a Forest Plot.

Comorbidities		HR (95% CI)	P value
	No hypertension	Reference	
	Model I, <0.5y	1.35 (1.27 – 143)	<0.0001
Hypertension	Model II, <0.5 - <3y	1.72 (1.56 – 1.90)	<0.0001
	Model III, 3 - <6y	1.78 (1.61 – 1.95)	<0.0001
	Model IV, >6y	1.74 (1.54 – 1.97)	<0.0001
	No CAD	Reference	
	Model I, <0.5y	1.24 (1.17 – 1.32)	<0.0001
CAD	Model II, <0.5 - <3y	1.51 (1.36 – 1.67)	<0.0001
	Model III, 3 - <6y	1.65 (1.51 – 1.80)	<0.0001
	Model IV, >6y	1.75 (1.59 – 1.93)	<0.0001
	No atrial fibrillation	Reference	
	Model I, <0.5y	1.25 (1.18 – 1.33)	<0.0001
Atrial fibrillation	Model II, <0.5 - <3y	1.54 (1.39 – 1.69)	<0.0001
	Model III, 3 - <6y	1.73 (1.60 – 1.89)	<0.0001
	Model IV, >6y	1.92 (1.70 – 2.16)	<0.0001
	No VHD	Reference	
	Model I, <0.5y	1.15 (1.07 – 1.24)	0.0004
VHD	Model II, <0.5 - <3y	1.30 (1.14 – 1.48)	<0.0001
	Model III, 3 - <6y	1.38 (1.23 – 1.54)	<0.0001
	Model IV, >6y	1.43 (1.20 – 1.70)	<0.0001
	No stroke	Reference	
	Model I, <0.5y	1.29 (1.19 – 1.40)	<0.0001
Stroke	Model II, <0.5 - <3y	1.57 (1.37 – 1.80)	<0.0001
	Model III, 3 - <6y	1.50 (1.31 – 1.71)	<0.0001
	Model IV, >6y	1.35 (1.06 – 1.71)	0.0136
	No hyperlipidemia	Reference	
	Model I, <0.5y	1.29 (1.22 – 1.37)	<0.0001
Hyperlipidemia	Model II, <0.5 - <3y	1.60 (1.46 – 1.77)	<0.0001
	Model III, 3 - <6y	1.68 (1.52 – 1.85)	<0.0001
	Model IV, >6y	1.68 (1.48 – 1.85)	<0.0001
	No hyperlipidemia	Reference	
	Model I, <0.5y	0.98 (0.92 – 1.06)	0.6604
DM	Model II, <0.5 - <3y	0.99 (0.88 – 1.12)	0.9195
	Model III, 3 - <6y	1.11 (1.01 – 1.21)	0.0026
	Model IV, >6y	1.27 (1.16 – 1.39)	<0.0001
	No obesity	Reference	
	Model I, <0.5y	0.89 (0.82 – 0.96)	0.0020
Obesity	Model II, <0.5 - <3y	0.83 (0.73 – 0.94)	0.0035
	Model III, 3 - <6y	0.98 (0.84 – 1.16)	0.8451
	Model IV, >6y	1.24 (0.89 – 1.71)	0.2027
	No CAD	Reference	
	Model I, <0.5y	1.35 (1.27 – 1.73)	<0.0001
CKD	Model II, <0.5 - <3y	1.74 (1.58 – 1.92)	<0.0001
	Model III, 3 - <6y	1.89 (1.69 – 2.12)	<0.0001
	Model IV, >6y	1.97 (1.60 – 2.42)	<0.0001
	No anemia	Reference	
	Model I, <0.5y	1.28 (1.15 – 1.43)	<0.0001
Anemia	Model II, <0.5 - <3y	1.58 (1.33 – 1.89)	<0.0001
	Model III, 3 - <6y	1.66 (1.43 – 1.94)	<0.0001
	Model IV, >6y	1.67 (1.29 – 2.17)	0.0001
	No CLC	Reference	
	Model I, <0.5y	1.25 (1.18 – 1.33)	<0.0001
CLC	Model II, <0.5 - <3y	1.48 (1.33 – 1.62)	<0.0001
	Model III, 3 - <6y	1.34 (1.19 – 1.51)	<0.0001
	Model IV, >6y	1.15 (0.92 – 1.44)	0.2307
	No GI/hepatic	Reference	
	Model I, <0.5y	1.29 (1.19 – 1.38)	<0.0001
GI/hepatic	Model II, <0.5 - <3y	1.54 (1.37 – 1.74)	<0.0001
	Model III, 3 - <6y	1.41 (1.27 – 1.58)	<0.0001
	Model IV, >6y	1.21 (1.02 – 1.44)	0.0317
	No cancer	Reference	
	Model I, <0.5y	1.37 (1.28 – 1.46)	<0.0001
Cancer	Model II, <0.5 - <3y	1.75 (1.57 – 1.94)	<0.0001
	Model III, 3 - <6y	1.71 (1.57 – 1.87)	<0.0001
	Model IV, >6y	1.57 (1.39 – 1.78)	<0.0001
	No rheum	Reference	
	Model I, <0.5y	1.23 (1.16 – 1.30)	<0.0001
Rheum	Model II, <0.5 - <3y	1.46 (1.32 – 1.61)	<0.0001
	Model III, 3 - <6y	1.51 (1.39 – 1.64)	<0.0001
	Model IV, >6y	1.51 (1.35 – 1.70)	<0.0001
	No neurological	Reference	
	Model I, <0.5y	1.34 (1.23 – 1.45)	<0.0001
Neurological	Model II, <0.5 - <3y	1.67 (1.46 – 1.89)	<0.0001
	Model III, 3 - <6y	1.57 (1.36 – 1.81)	<0.0001
	Model IV, >6y	1.38 (1.04 – 1.83)	0.0277
	No psychiatric	Reference	
	Model I, <0.5y	1.10 (1.03 – 1.17)	0.0066
Psychiatric	Model II, <0.5 - <3y	1.18 (1.06 – 1.32)	0.0035
	Model III, 3 - <6y	1.18 (1.08 – 1.29)	0.0003
	Model IV, >6y	1.16 (1.03 – 1.31)	0.0170

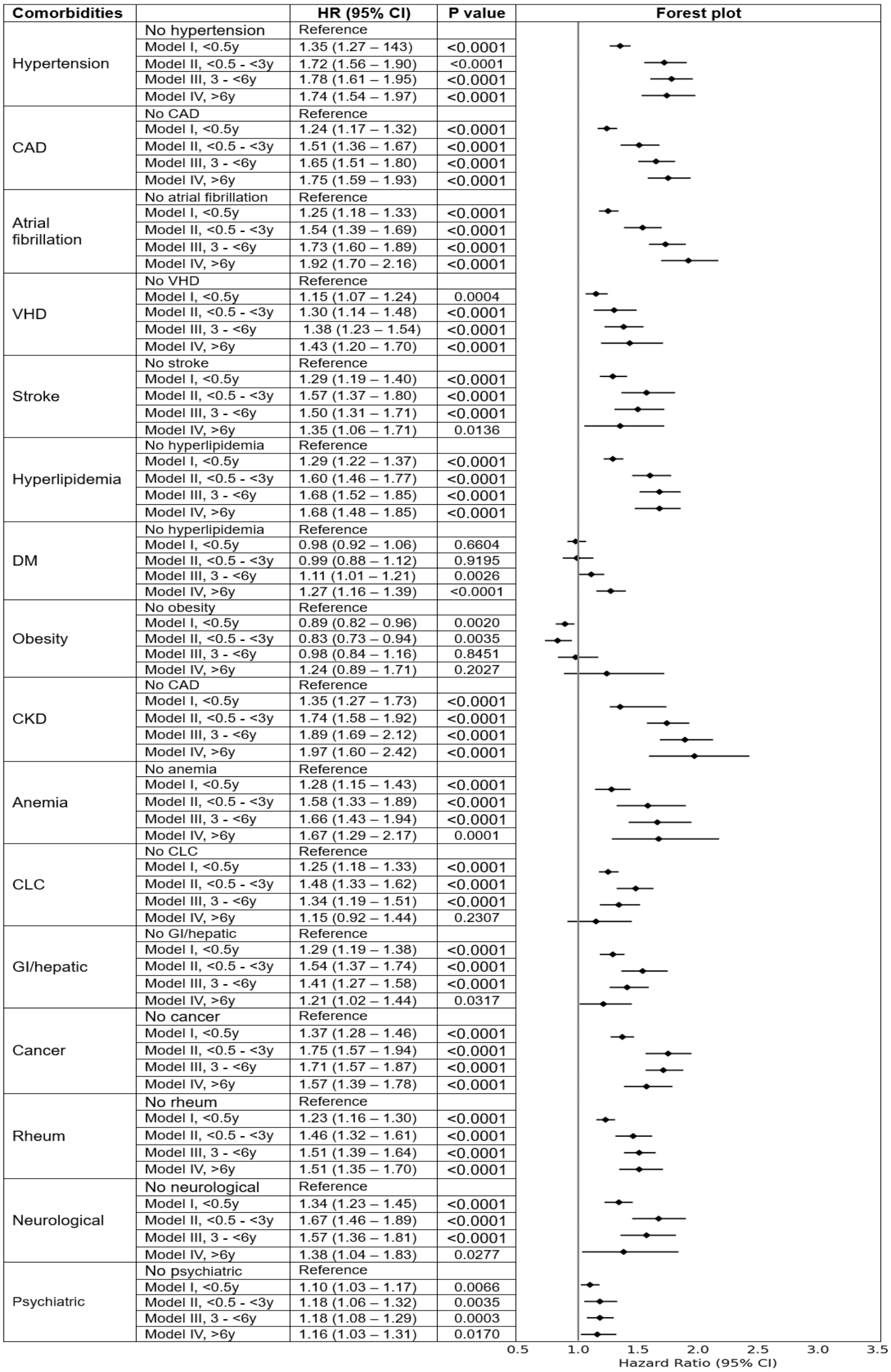

Model I: Multivariable Cox regression model estimating mortality based on preadmission comorbidity duration of < 0.5 years.
Model II: Multivariable Cox regression model estimating mortality based on preadmission comorbidity duration of 0.5 to < 3 years.
Model III: Multivariable Cox regression model estimating mortality based on preadmission comorbidity duration of 3 to < 6 years.
Model IV: Multivariable Cox regression model estimating mortality based on preadmission comorbidity duration of ≥ 6 years.

Most comorbidities demonstrated an increase in effect size (HR) as the duration extended from < 0.5 years to 0.5 to < 3 years, followed by either a slower increase, a plateau, or even a decline once the comorbidity duration reached 3 years or more.

Abbreviations: CAD, coronary artery disease; CLC, chronic lung condition; CKD, chronic kidney disease ≥ stage 3; DM, diabetes mellitus; GI/hepatic, gastrointestinal/hepatic conditions; Neurological, non-stroke neurological condition; Psychiatric, psychiatric condition; Rheum, rheumatological condition; VHD, valvular heart disease.

Supplement Figure 5A. Adjusted Hazard Ratios and 95% Confidence Intervals for Mortality by Comorbidity Duration in Patients from Rural Counties, Displayed as a Forest Plot.

Comorbidities		HR (95% CI)	P value
Hypertension	No hypertension	Reference	
	Model I, <0.5y	1.35 (1.27 – 1.43)	<0.0001
	Model II, <0.5 - <3y	1.72 (1.55 – 1.90)	<0.0001
	Model III, 3 - <6y	1.70 (1.54 – 1.88)	<0.0001
	Model IV, >6y	1.59 (1.39 – 1.80)	<0.0001
CAD	No CAD	Reference	
	Model I, <0.5y	1.21 (1.14 – 1.29)	<0.0001
	Model II, <0.5 - <3y	1.42 (1.28 – 1.58)	<0.0001
	Model III, 3 - <6y	1.45 (1.35 – 1.60)	<0.0001
	Model IV, >6y	1.47 (1.32 – 1.64)	<0.0001
Atrial fibrillation	No atrial fibrillation	Reference	
	Model I, <0.5y	1.23 (1.16 – 1.30)	<0.0001
	Model II, <0.5 - <3y	1.47 (1.33 – 1.62)	<0.0001
	Model III, 3 - <6y	1.58 (1.44 – 1.73)	<0.0001
	Model IV, >6y	1.65 (1.44 – 1.89)	<0.0001
VHD	No VHD	Reference	
	Model I, <0.5y	1.13 (1.03 – 1.23)	0.0072
	Model II, <0.5 - <3y	1.24 (1.07 – 1.44)	0.0037
	Model III, 3 - <6y	1.24 (1.09 – 1.41)	0.0010
	Model IV, >6y	1.21 (0.98 – 1.48)	0.0063
Stroke	No stroke	Reference	
	Model I, <0.5y	1.19 (1.08 – 1.30)	0.0003
	Model II, <0.5 - <3y	1.35 (1.16 – 1.57)	<0.0001
	Model III, 3 - <6y	131 (1.13 – 1.51)	0.0002
	Model IV, >6y	1.22 (0.93 – 1.58)	0.1445
Hyperlipidemia	No hyperlipidemia	Reference	
	Model I, <0.5y	1.29 (1.22 – 1.36)	<0.0001
	Model II, <0.5 - <3y	1.56 (1.41 – 1.72)	<0.0001
	Model III, 3 - <6y	1.47 (1.34 – 1.62)	<0.0001
	Model IV, >6y	1.31 (1.14 – 1.49)	<0.0001
DM	No hyperlipidemia	Reference	
	Model I, <0.5y	0.99 (0.93 – 4.07)	0.8798
	Model II, <0.5 - <3y	1.00 (0.89 – 1.13)	0.9728
	Model III, 3 - <6y	1.06 (0.97 – 1.16)	0.1862
	Model IV, >6y	1.14 (1.03 – 1.26)	0.0085
Obesity	No obesity	Reference	
	Model I, <0.5y	0.84 (0.76 – 0.90)	<0.0001
	Model II, <0.5 - <3y	0.74 (0.65 – 0.85)	<0.0001
	Model III, 3 - <6y	0.89 (0.74 – 1.07)	0.2091
	Model IV, >6y	1.15 (0.79 – 1.67)	0.4746
CKD	No CAD	Reference	
	Model I, <0.5y	1.40 (1.30 – 1.50)	<0.0001
	Model II, <0.5 - <3y	1.81 (1.62 – 2.03)	<0.0001
	Model III, 3 - <6y	1.76 (1.53 – 2.02)	<0.0001
	Model IV, >6y	1.59 (1.22 – 2.06)	0.0006
Anemia	No anemia	Reference	
	Model I, <0.5y	130 (1.19 – 1.43)	<0.0001
	Model II, <0.5 - <3y	1.62 (1.39 – 1.88)	<0.0001
	Model III, 3 - <6y	1.63 (1.43 – 1.86)	<0.0001
	Model IV, >6y	1.67 (1.26 – 1.940	<0.0001
CLC	No CLC	Reference	
	Model I, <0.5y	1.18 (1.11 – 1.26)	<0.0001
	Model II, <0.5 - <3y	1.33 (1.20 – 1.48)	<0.0001
	Model III, 3 - <6y	1.23 (1.08 – 1.39)	0.0015
	Model IV, >6y	1.07 (0.84 – 1.36)	0.5698
GI/hepatic	No GI/hepatic	Reference	
	Model I, <0.5y	1.21 (1.12 – 1.30)	<0.0001
	Model II, <0.5 - <3y	1.37 (1.21 – 1.56)	<0.0001
	Model III, 3 - <6y	1.22 (1.09 – 1.38)	0.0008
	Model IV, >6y	1.03 (0.85 – 1.25)	0.7778
Cancer	No cancer	Reference	
	Model I, <0.5y	1.30 (1.22 – 1.40)	<0.0001
	Model II, <0.5 - <3y	1.59 (1.41 – 1.790	<0.0001
	Model III, 3 - <6y	1.47 (1.34 – 1.62)	<0.0001
	Model IV, >6y	1.27 (1.10 – 1.48)	0.0012
Rheum	No rheum	Reference	
	Model I, <0.5y	1.19 (1.12 – 126)	<0.0001
	Model II, <0.5 - <3y	1.37 (1.23 – 1.51)	<0.0001
	Model III, 3 - <6y	1.38 (1.27 – 1.51)	<0.0001
	Model IV, >6y	1.36 (1.20 – 1.53)	<0.0001
Neurological	No neurological	Reference	
	Model I, <0.5y	1.51 (1.39 – 1.63)	<0.0001
	Model II, <0.5 - <3y	2.05 (1.80 – 2.32)	<0.0001
	Model III, 3 - <6y	1.84 (1.59 – 2.14)	<0.0001
	Model IV, >6y	1.50 (1.13 – 2.00)	0.0055
Psychiatric	No psychiatric	Reference	
	Model I, <0.5y	1.08 (1.01 – 1.15)	0.0238
	Model II, <0.5 - <3y	1.14 (1.02 – 1.27)	0.0207
	Model III, 3 - <6y	1.10 (1.01 – 1.21)	0.0315
	Model IV, >6y	1.05 (0.93 – 1.18)	0.4485

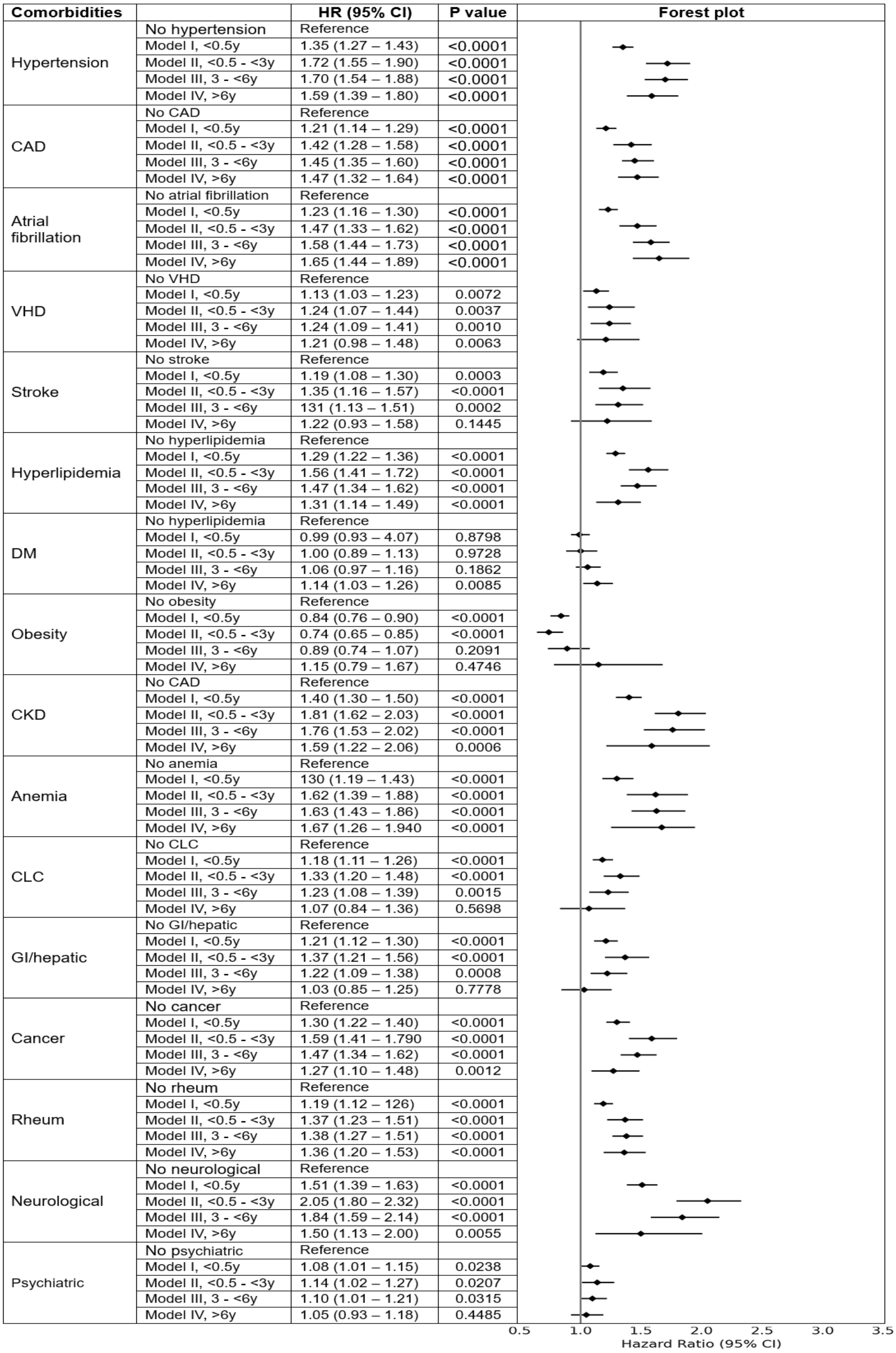

Model I: Multivariable Cox regression model estimating mortality based on preadmission comorbidity duration of < 0.5 years.
Model II: Multivariable Cox regression model estimating mortality based on preadmission comorbidity duration of 0.5 to < 3 years.
Model III: Multivariable Cox regression model estimating mortality based on preadmission comorbidity duration of 3 to < 6 years.
Model IV: Multivariable Cox regression model estimating mortality based on preadmission comorbidity duration of ≥ 6 years.

Most comorbidities demonstrated an increase in effect size (HR) as the duration extended from < 0.5 years to 0.5 to < 3 years, followed by either a slower increase, a plateau, or even a decline once the comorbidity duration reached 3 years or more.

Abbreviations: CAD, coronary artery disease; CLC, chronic lung condition; CKD, chronic kidney disease ≥ stage 3; DM, diabetes mellitus; GI/hepatic, gastrointestinal/hepatic conditions; Neurological, non-stroke neurological condition; Psychiatric, psychiatric condition; Rheum, rheumatological condition; VHD, valvular heart disease.

Supplement Figure 5B. Adjusted Hazard Ratios and 95% Confidence Intervals for Mortality by Comorbidity Duration in Patients from Urban Counties, Displayed as a Forest Plot.

Comorbidities		HR (95% CI)	P value	Forest plot
Hypertension	No hypertension	Reference		
	Model I, <0.5y	1.37 (1.30 – 1.44)	<0.0001	
	Model II, <0.5 - <3y	1.77 (1.62 – 1.93)	<0.0001	
	Model III, 3 - <6y	1.79 (1.64 – 1.94)	<0.0001	
	Model IV, >6y	1.70 (1.51 – 1.90)	<0.0001	
CAD	No CAD	Reference		
	Model I, <0.5y	1.23 (1.16 – 1.30)	<0.0001	
	Model II, <0.5 - <3y	1.46 (1.33 – 1.61)	<0.0001	
	Model III, 3 - <6y	1.56 (1.44 – 1.68)	<0.0001	
	Model IV, >6y	1.61 (1.47 – 1.76)	<0.0001	
Atrial fibrillation	No atrial fibrillation	Reference		
	Model I, <0.5y	1.24 (1.18 – 1.30)	<0.0001	
	Model II, <0.5 - <3y	1.49 (1.37 – 1.63)	<0.0001	
	Model III, 3 - <6y	1.60 (1.48 – 1.73)	<0.0001	
	Model IV, >6y	1.66 (1.49 – 1.85)	<0.0001	
VHD	No VHD	Reference		
	Model I, <0.5y	1.16 (1.08 – 1.25)	<0.0001	
	Model II, <0.5 - <3y	1.32 (1.17 – 1.48)	<0.0001	
	Model III, 3 - <6y	1.38 (1.24 – 1.53)	<0.0001	
	Model IV, >6y	1.41 (1.20 – 1.66)	<0.0001	
Stroke	No stroke	Reference		
	Model I, <0.5y	1.27 (1.19 – 1.37)	<0.0001	
	Model II, <0.5 - <3y	1.54 (1.37 – 1.73)	<0.0001	
	Model III, 3 - <6y	1.49 (1.33 – 1.67)	<0.0001	
	Model IV, >6y	1.37 (1.11 – 1.69)	0.0030	
Hyperlipidemia	No hyperlipidemia	Reference		
	Model I, <0.5y	1.26 (1.20 – 1.32)	<0.0001	
	Model II, <0.5 - <3y	1.53 (1.41 – 1.67)	<0.0001	
	Model III, 3 - <6y	1.65 (1.52 – 1.80)	<0.0001	
	Model IV, >6y	1.73 (1.53 – 1.95)	<0.0001	
DM	No hyperlipidemia	Reference		
	Model I, <0.5y	0.97 (0.91 – 1.04)	0.4094	
	Model II, <0.5 - <3y	0.98 (0.88 – 1.09)	0.6493	
	Model III, 3 - <6y	1.10 (1.01 – 1.19)	0.0240	
	Model IV, >6y	1.27 (1.17 – 1.38)	<0.0001	
Obesity	No obesity	Reference		
	Model I, <0.5y	0.90 (0.84 – 0.97)	0.0047	
	Model II, <0.5 - <3y	0.85 (0.76 – 0.960	0.0061	
	Model III, 3 - <6y	0.97 (0.82 – 1.14)	0.6940	
	Model IV, >6y	1.15 (0.82 – 1.61)	0.4114	
CKD	No CAD	Reference		
	Model I, <0.5y	1.36 (1.29 – 1.44)	<0.0001	
	Model II, <0.5 - <3y	1.76 (1.60 – 1.92)	<0.0001	
	Model III, 3 - <6y	1.83 91.65 – 2.04)	<0.0001	
	Model IV, >6y	1.82 (1.50 – 2.21)	<0.0001	
Anemia	No anemia	Reference		
	Model I, <0.5y	1.29 (1.18 – 1.40)	<0.0001	
	Model II, <0.5 - <3y	1.57 (1.37 – 1.81)	<0.0001	
	Model III, 3 - <6y	1.58 (1.39 – 1.78)	<0.0001	
	Model IV, >6y	1.50 (1.22 – 1.84)	0.0001	
CLC	No CLC	Reference		
	Model I, <0.5y	1.22 (1.15 – 1.29)	<0.0001	
	Model II, <0.5 - <3y	1.42 (1.30 – 1.55)	<0.0001	
	Model III, 3 - <6y	1.37 (1.23 – 1.51)	<0.0001	
	Model IV, >6y	1.26 (1.04 – 1.52)	0.0189	
GI/hepatic	No GI/hepatic	Reference		
	Model I, <0.5y	1.24 (1.16 – 1.33)	<0.0001	
	Model II, <0.5 - <3y	1.47 (1.31 – 1.64)	<0.0001	
	Model III, 3 - <6y	1.41 (1.28 – 1.55)	<0.0001	
	Model IV, >6y	1.29 (1.11 – 1.50)	0.0010	
Cancer	No cancer	Reference		
	Model I, <0.5y	1.26 (1.19 – 1.34)	<0.0001	
	Model II, <0.5 - <3y	1.52 (1.38 – 1.68)	<0.0001	
	Model III, 3 - <6y	1.52 (1.40 – 1.65)	<0.0001	
	Model IV, >6y	1.45 (1.29 – 1.62)	<0.0001	
Rheum	No rheum	Reference		
	Model I, <0.5y	1.20 (1.14 – 1.27)	<0.0001	
	Model II, <0.5 - <3y	1.39 (1.27 – 1.52)	<0.0001	
	Model III, 3 - <6y	1.38 (1.28 – 1.49)	<0.0001	
	Model IV, >6y	1.32 (1.19 – 1.47)	<0.0001	
Neurological	No neurological	Reference		
	Model I, <0.5y	1.35 (1.26 – 1.44)	<0.0001	
	Model II, <0.5 - <3y	1.67 (1.49 – 1.87)	<0.0001	
	Model III, 3 - <6y	1.49 (1.31 – 1.69)	<0.0001	
	Model IV, >6y	1.22 (0.96 – 1.56)	0.1005	
Psychiatric	No psychiatric	Reference		
	Model I, <0.5y	1.07 (1.01 – 1.14)	0.0267	
	Model II, <0.5 - <3y	1.13 (1.02 – 1.25)	0.0155	
	Model III, 3 - <6y	1.13 (1.06 – 1.24)	0.0009	
	Model IV, >6y	1.15 (1.04 – 1.26)	0.0074	

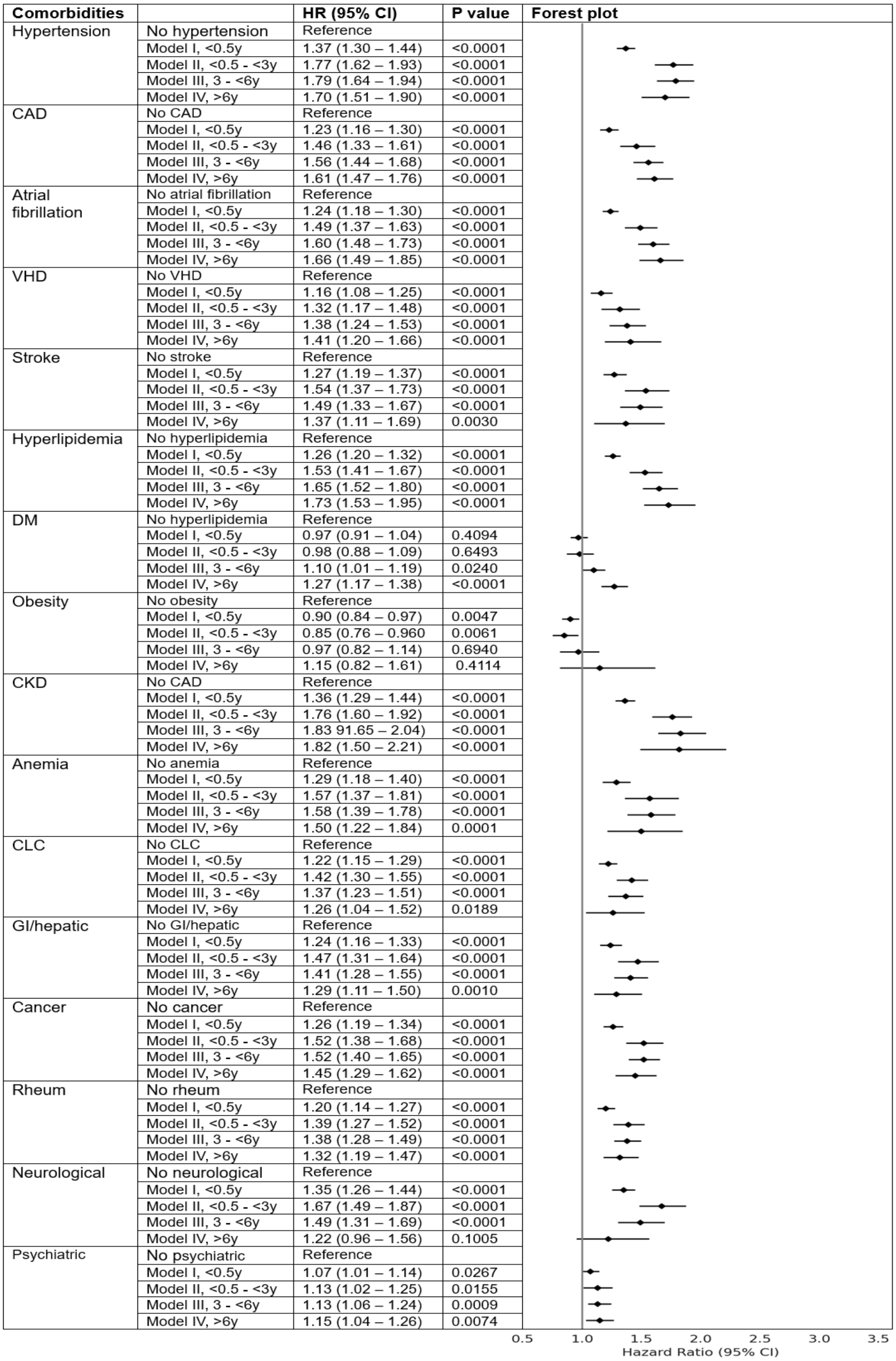

Model I: Multivariable Cox regression model estimating mortality based on preadmission comorbidity duration of < 0.5 years.
Model II: Multivariable Cox regression model estimating mortality based on preadmission comorbidity duration of 0.5 to < 3 years.
Model III: Multivariable Cox regression model estimating mortality based on preadmission comorbidity duration of 3 to < 6 years.
Model IV: Multivariable Cox regression model estimating mortality based on preadmission comorbidity duration of ≥ 6 years.

Most comorbidities demonstrated an increase in effect size (HR) as the duration extended from < 0.5 years to 0.5 to < 3 years, followed by either a slower increase, a plateau, or even a decline once the comorbidity duration reached 3 years or more.

Abbreviations: CAD, coronary artery disease; CLC, chronic lung condition; CKD, chronic kidney disease ≥ stage 3; DM, diabetes mellitus; GI/hepatic, gastrointestinal/hepatic conditions; Neurological, non-stroke neurological condition; Psychiatric, psychiatric condition; Rheum, rheumatological condition; VHD, valvular heart disease.

Supplement Figure 6. Association between comorbidities and mortality across heart failure severity defined by GWTG score

	GWTG 1			GWTG 2			GWTG 3 or more		
**Comorbidities**	**Events (%)/n=**	**HR (95% CI), P value**	**Forest Plot**	**Events (%)/n=**	**HR (95% CI), P value**	**Forest Plot**	**Events (%)/n=**	**HR (95% CI), P value**	**Forest Plot**
**Cardiometabolic-renal-anemia**									
Hypertension	339 (42)/809	1.46 (1.21 - 1.77), 0.0001		3384 (67)/5060	1.45 (1.35 - 1.55), <0.0001		762 (82)/929	1.37 (1.17 - 1.61), <0.001	
CAD	217 (48)/448	1.38 (1.07 - 1.78), 0.0134		2238 (71)/3140	1.38 (1.29 - 1.47), <0.0001		482 (83)/581	1.42 (1.25 - 1.62), <0.0001	
Atrial fibrillation	177 (45)/393	1.25 (1.04 - 1.51), 0.0179		2383 (68)/3484	1.15 (1.08 - 1.22), <0.0001		614 (82)/753	1.02 (0.90 - 1.17), 0.6853	
Valve disease	61 (34)/178	0.99 (0.76 - 1.30), 0.9635		866 (67)/1294	1.10 (1.03 - 1.19), 0.0088		218 (85)/255	1.27 (1.09 - 1.48), 0.0018	
Stroke	60 (43)/141	1.20 (0.92 - 1.58), 0.1871		690 (71)/975	1.34 (1.24 - 1.44), <0.0001		136 (87)/157	1.37 (1.14 - 1.64), 0.0008	
Hyperlipidemia	298 (41)/732	1.27 (1.05 - 1.53), 0.0121		3092 (66)/4664	1.31 (1.23 - 1.40), <0.0001		697 (82)/855	1.19 (1.03 - 1.37), 0.0201	
Diabetes mellitus	261 (46)/569	1.20 (1.00 - 1.42), 0.0438		1993 (68)/2923	1.19 (1.12 - 1.26), <0.0001		421 (82)/515	1.24 (1.09 - 1.41), 0.0010	
Obesity	76 (25)/301	0.72 (0.57 - 0.93), 0.0114		828 (54)/1533	0.94 (0.87 - 1.02), 0.1446		175 (76)/230	1.11 (0.94 - 1.63), 0.2365	
CKD	74 (42)/175	1.38 (1.07 - 1.78), 0.0134		1218 (72)/1692	1.38 (1.29 - 1.47), <0.0001		427 (85)/504	1.42 (1.25 - 1.62), <0.0001	
Anemia	53 (56)/94	1.61 (1.21 - 2.14), 0.0011		613 (76)/780	1.45 (1.33 - 1.58), <0.0001		152 (89)/171	1.25 (1.05 - 1.48), 0.0134	
**Other system-based conditions**									
Chronic lung conditions	65 (45)/146	1.37 (1.05 - 1.78), 0.0184		879 (70)/1257	1.31 (1.22 - 1.41), <0.0001		192 (81)/238	1.08 (0.92 - 1.27), 0.3195	
Gastrointestinal and hepatic	81 (46)/175	1.41 (1.11 - 1.79), 0.0046		889 (69)/1289	1.24 (1.15 - 1.34), <0.0001		260 (83)/312	1.26 (1.09 - 1.46), 0.0016	
Oncological conditions	107 (50)/216	1.47 (1.19 - 1.83), 0.0004		1348 (70)/1926	1.30 (1.22 - 1.38), <0.0001		351 (85)/412	1.36 (1.19 - 1.56), <0.0001	
Rheumatological	43 (41)/104	1.10 (0.80 - 1.51), 0.5462		497 (65)/770	1.11 (1.01 - 1.22), 0.0262		90 (83)/108	1.22 (0.98 - 1.52), 0.0741	
Neurological conditions	39 (51)/76	2.13 (1.66 - 2.73), <0.0001		290 (70)/417	1.50 (1.39 - 1.62), <0.0001		51 (80)/64	1.15 (0.97 - 1.35), 0.1073	
Psychiatric conditions	53 (35)/151	1.15 (0.86 - 1.53), 0.3479		367 (56)/618	1.01 (0.91 - 1.13), 0.8529		73 (82)/89	1.28 (1.01 - 1.63), 0.0428	
			0.0 1.0 2.5			0.0 1.0 2.5			0.0 1.0 2.5

Forest plots show hazard ratios (HRs) with 95% confidence intervals (CIs) for mortality associated with individual comorbidities across GWTG 1, GWTG 2, and GWTG ≥3 severity categories. Most comorbidities were linked to higher mortality, though strength varied; neurological conditions showed the strongest association in GWTG 1–2, while obesity showed a neutral or protective effect.

Supplement Table 5. STROBE Check List (22 items)

	Item No	Recommendation	Page
**Title and abstract**	1	(*a*) Indicate the study's design with a commonly used term in the title or the abstract	**2 and 3**
		(*b*) Provide in the abstract an informative and balanced summary of what was done and what was found	
**Introduction**			
Background/rationale	2	Explain the scientific background and rationale for the investigation being reported	**4**
Objectives	3	State specific objectives, including any prespecified hypotheses	**6, 7**
**Methods**			
Study design	4	Present key elements of study design early in the paper	**5**
Setting	5	Describe the setting, locations, and relevant dates, including periods of recruitment, exposure, follow-up, and data collection	**5 – 8**
Participants	6	*(a)* *Cohort study*—Give the eligibility criteria, and the sources and methods of selection of participants. *(b)* Describe methods of follow-up<br>*Case-control study*—Give the eligibility criteria, and the sources and methods of case ascertainment and control selection. Give the rationale for the choice of cases and controls<br>*Cross-sectional study*—Give the eligibility criteria, and the sources and methods of selection of participants	**5**
		(*b*) *Cohort study*—For matched studies, give matching criteria and number of exposed and unexposed<br>*Case-control study*—For matched studies, give matching criteria and the number of controls per case	
Variables	7	Clearly define all outcomes, exposures, predictors, potential confounders, and effect modifiers. Give diagnostic criteria, if applicable	**5-8**
Data sources/ measurement	8*	For each variable of interest, give sources of data and details of methods of assessment (measurement). Describe comparability of assessment methods if there is more than one group	**5-8**
Bias	9	Describe any efforts to address potential sources of bias	**14**
Study size	10	Explain how the study size was arrived at	not applicable
Quantitative variables	11	Explain how quantitative variables were handled in the analyses. If applicable, describe which groupings were chosen and why	Not applicable
Statistical methods	12	(*a*) Describe all statistical methods, including those used to control for confounding	**7 and 8**
		(*b*) Describe any methods used to examine subgroups and interactions	
		(*c*) Explain how missing data were addressed	
		(*d*) *Cohort study*—If applicable, explain how loss to follow-up was addressed<br>*Case-control study*—If applicable, explain how matching of cases and controls was addressed<br>*Cross-sectional study*—If applicable, describe analytical methods taking account of sampling strategy	

(*e*) Describe any sensitivity analyses

Continued on next page

Results			
Participants	13*	(a) Report numbers of individuals at each stage of study—eg numbers potentially eligible, examined For eligibility, confirmed eligible, included in the study, completing follow-up, and analysed	**Supplement Fig 1**
		(b) Give reasons for non-participation at each stage	
		(c) Consider use of a flow diagram	**Supplement Fig 1**
Descriptive data	14*	(a) Give characteristics of study participants (eg demographic, clinical, social) and information on exposures and potential confounders	
		(b) Indicate number of participants with missing data for each variable of interest	**9**
		(c) *Cohort study*—Summarise follow-up time (eg, average and total amount)	**7**
Outcome data	15*	*Cohort study*—Report numbers of outcome events or summary measures over time	**7**
		Case-control study—Report numbers in each exposure category, or summary measures of exposure	
		Cross-sectional study—Report numbers of outcome events or summary measures	
Main results	16	(*a*) Give unadjusted estimates and, if applicable, confounder-adjusted estimates and their precision (eg, 95% confidence interval). Make clear which confounders were adjusted for and why they were included	**8**
		(*b*) Report category boundaries when continuous variables were categorized	
		(*c*) If relevant, consider translating estimates of relative risk into absolute risk for a meaningful time period	
Other analyses	17	Report other analyses done—eg analyses of subgroups and interactions, and sensitivity analyses	**7**
**Discussion**			
Key results	18	Summarise key results with reference to study objectives	**9 - 12**
Limitations	19	Discuss limitations of the study, taking into account sources of potential bias or imprecision. Discuss both direction and magnitude of any potential bias	**14, 15**
Interpretation	20	Give a cautious overall interpretation of results considering objectives, limitations multiplicity of analyses, results from similar studies, and other relevant evidence	**12, 13**
Generalisability	21	Discuss the generalisability (external validity) of the study results	**14, 15**
**Other information**			
Funding	22	Give the source of funding and the role of the funders for the present study and, if applicable, for the original study on which the present article is based	**16**

*Give information separately for cases and controls in case-control studies and, if applicable, for exposed and unexposed groups in cohort and cross-sectional studies.

Note: An Explanation and Elaboration article discusses each checklist item and gives methodological background and published examples of transparent reporting. The STROBE checklist is best used in conjunction with this article (freely available on the Web sites of PLoS Medicine at http://www.plosmedicine.org/, Annals of Internal Medicine at http://www.annals.org/, and Epidemiology at http://www.epidem.com/). Information on the STROBE Initiative is available at www.strobe-statement.org.
